# Encoding and Encryption via Dual‐Wavelength 3D Digital Light Processing

**DOI:** 10.1002/anie.1978756

**Published:** 2026-07-17

**Authors:** Finn Kröger, Michał P. Świątek, Eva Blasco

**Affiliations:** ^1^ Institute for Molecular Systems Engineering and Advanced Materials Heidelberg University Heidelberg Germany; ^2^ Institute of Organic Chemistry Heidelberg University Heidelberg Germany

**Keywords:** chromophore, digital light processing, encryption, photopolymer, spiropyran

## Abstract

Additive manufacturing of multi‐responsive materials is opening new opportunities in a wide range of applications, including information storage, security, and sensing. Herein, complex structures enabling both the encoding and encryption of information in three dimensions (3D) are achieved by combining photo‐ and pH‐responsive chromophores, spiropyran and phenolphthalein derivatives, respectively, within a single ink formulation. To this end, a multi‐wavelength digital light processing (DLP) system is employed to fabricate initially colorless structures using a 405 nm wavelength LED to trigger the photopolymerization process. In parallel, a 365 nm wavelength LED integrated into the same system can induce locally controlled isomerization of spiropyran to the colored merocyanine, enabling information encoding in 3D in a layer‐by‐layer fashion. The stored information can be reversibly encrypted on demand via pH variation. The pH‐responsive chromophore, phenolphthalein, enables modulation of optical contrast, rendering the encoded patterns temporarily unreadable. This work opens new pathways toward engineering multi‐responsive functionality in complex 3D architectures.

## Graphical abstract

Multi‐wavelength 3D DLP printing enables information encoding that can be encrypted on demand by applying orthogonal stimuli within a single material to trigger complementary responses. The 405 nm LED of the printer fabricates complex 3D geometries, while the 365 nm LED activates spiropyrans photochromism to program information into the structure. Phenolphthalein's pH‐responsiveness enables reversible encryption.

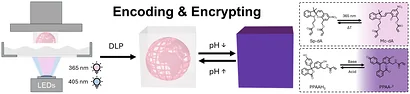

## 1 Introduction

Light‐responsive systems, particularly photochromic materials, have attracted sustained interest over decades due to their broad applicability in camouflage [1], optical switches [2], data storage [3, 4], or as warning systems [5, 6]. Central to their function are reversible processes such as *E–Z* isomerization and bond cleavage, which induce pronounced changes in absorption spectra and enable finely tunable optical properties [6, 7, 8, 9]. Among these systems, spiropyran (Sp) stands out as a highly versatile photochromic compound. The colorless Sp can undergo UV‐induced isomerization via heterolytic cleavage of the C_spiro–_O bond, yielding the zwitterionic colored merocyanine (Mc) [10, 11, 12, 13, 14, 15, 16]. Beyond photoactivation, the rich chemical tunability of Sp derivatives enables responsiveness to a wide range of external stimuli, including mechanical force [17, 18], temperature [19], metal ions [20], ultrasonic waves [21], and pH changes [19, 22], thereby establishing Sp as a powerful platform for responsive material design.

Recently, significant advances have been made in integrating spiropyran chromophores into additive manufacturing (AM), enabling the on‐demand fabrication of customizable 3D geometries [23]. Among light‐based AM techniques, digital light processing (DLP) represents a particularly powerful approach owing to its high fabrication precision and outstanding surface finish [24]. For example, Loos and coworkers demonstrated that Sp can be incorporated into dynamic disulfides and β‐hydroxy ester‐based ink formulations. The 3D DLP‐printed structures could be reversible isomerized via irradiation with UV‐ or visible (Vis)‐light, demonstrating the photochromic response of Sp by switching the color between colorless and orange [25]. In a previous work from our group, we covalently incorporated an Sp‐derivative into a 3D DLP printable ink formulation and demonstrated the photochromic as well as mechanochromic properties of the 3D structures [26]. Pisignano and co‐workers further demonstrated the photochromic properties of Sp in a 3D DLP printed staircase structure by selectively applying a laser, inducing a color change from yellow to purple individually for every “step,” thereby enabling the storage of binary information [27]. Despite their well‐established single‐stimulus responsiveness, unlocking the full potential of Sp requires their integration into complex 3D architectures capable of multi‐responsive functionality, thus providing a versatile platform for increased functional complexity. In particular, combining multiple orthogonal stimuli within a single material to elicit complimentary responses has gained considerable interest [28, 29, 30, 31, 32]. Nevertheless, the development of multi‐responsive, DLP‐printed systems incorporating organic photochromic materials remains an emerging and promising yet still underexplored direction.

**FIGURE 1:**
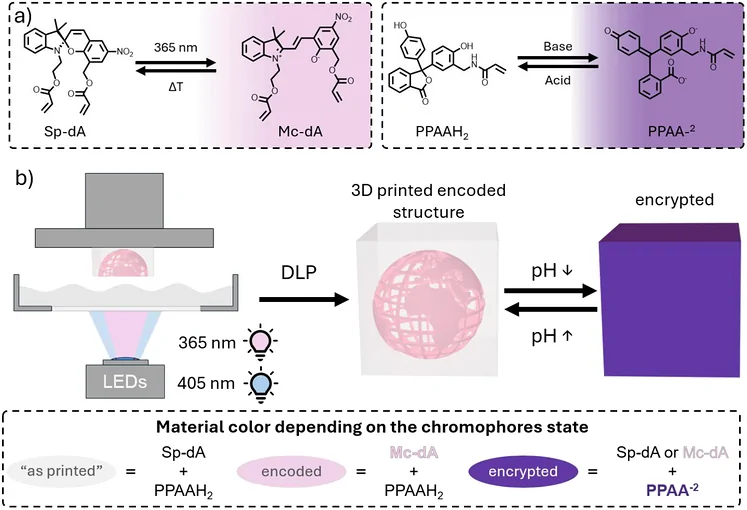
(a) Dual‐system based on a photochromic compound (spiropyran) and a pH‐responsive chromophore (phenolphthalein) employed in this work. (b) Schematic representation of the approach: a multi‐wavelength DLP printer is employed for the fabrication (405 nm wavelength LED) and encoding of optical information via light‐induced isomerization of spiropyran (365 nm wavelength LED). The programmed information in the 3D printed structure can be encrypted on demand by activating the pH‐responsive unit (phenolphthalein).

Here, we present an approach for the fabrication of multi‐responsive complex structures that enable both the encoding and encryption of information in 3D. Information is encoded via spatially controlled photochromism, while encryption is achieved through pH‐triggered modulation of optical contrast, rendering the encoded patterns temporarily unreadable. The deletion of the encoded information is enabled by thermally induced deactivation of the photochromic functionality. To realize this concept, a tailored ink formulation was developed incorporating spiropyran diacrylate (Sp‐dA) as a photochromic unit for light‐induced encoding and phenolphthalein acrylamide (PPAAH_2_) as a pH‐responsive chromophore for reversible encryption. A multi‐wavelength DLP printer was employed, equipped with a 405 and 365 nm wavelength LED selected for fabrication and optical encoding, respectively (Figure 1). Importantly, the versatility of the dual system is demonstrated in complex 3D architectures containing arbitrarily encoded information that can be encrypted on demand.

## 2 Results and Discussion

### 2.1 Light‐Induced Encoding in 3D Structures

First, the development of an ink formulation enabling the fabrication of colorless 3D structures, into which information can be encoded during the printing process, was targeted. To this end, Sp was selected as a suitable chromophore due to its reversible isomerization from the colorless Sp to a colored Mc form upon UV‐irradiation. A key consideration in this context is the selection of appropriate irradiation wavelengths, as they must enable both the selective printing and programming process. Specifically, the wavelength used for polymerization must efficiently initiate curing while avoiding undesired isomerization of Sp to Mc to generate a transparent material, whereas the wavelength used for programming must selectively trigger the photoisomerization. Based on these requirements, a multi‐wavelength DLP printer was employed, and the 405 nm wavelength LED was selected for the DLP printing process, while a 365 nm wavelength LED was used for encoding, as it efficiently induces the isomerization from the colorless Sp to the colored Mc form. This setup allows, immediately after printing of each layer, spatially resolved encoding. To incorporate the photochromic Sp covalently into 3D DLP printed structures enabling a reliable response, it was equipped with acrylates to yield the Sp‐dA via a synthesis route previously established in the group (see supporting information (SI), Section 3: Synthesis) [26].

**FIGURE 2:**
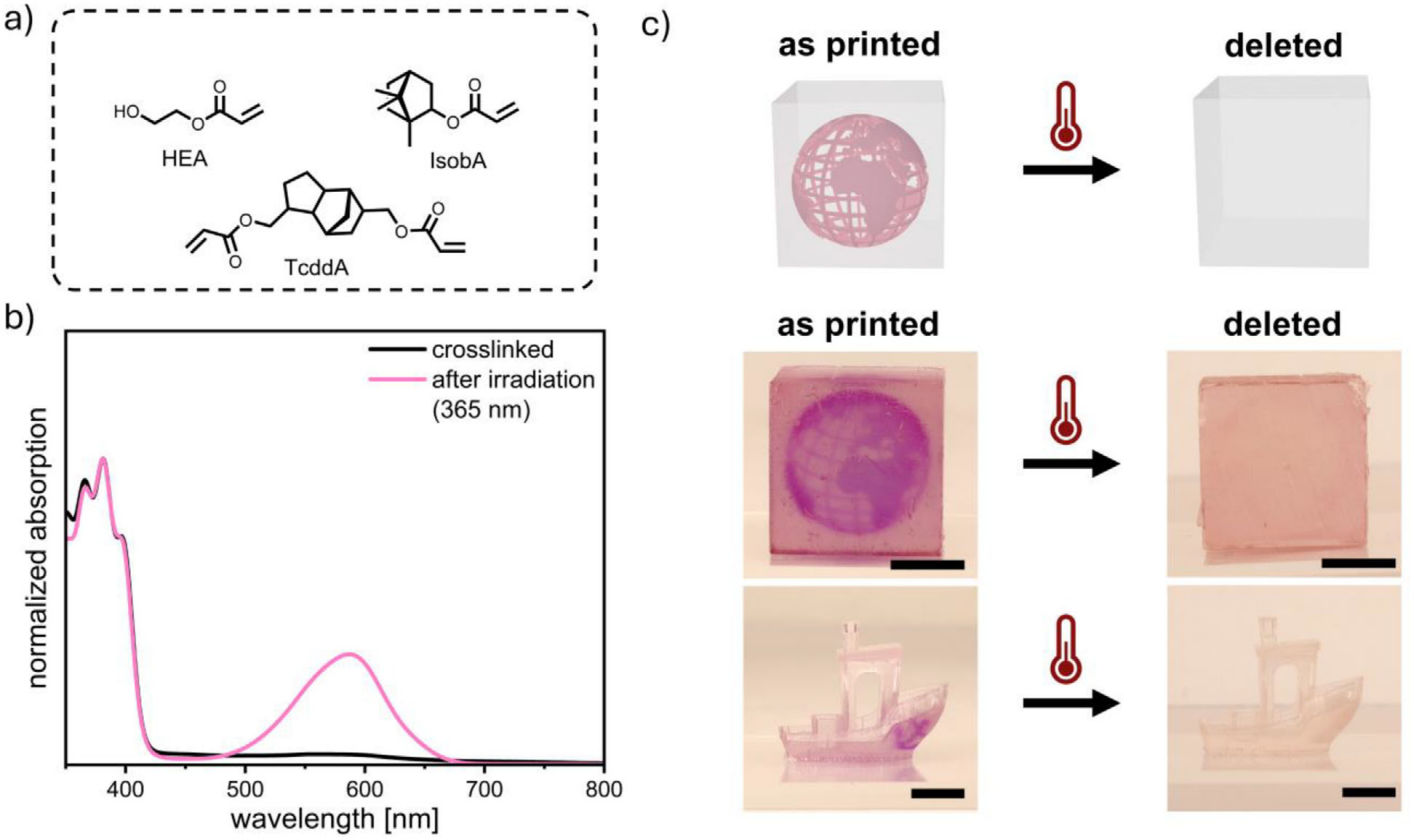
(a) Chemical structure of monomers and crosslinkers employed in the printable ink formulation. (b) UV/Vis spectra of a film from the optimized ink formulation are shown before (colorless) and after irradiation (purple) demonstrating the photochromic behavior. (c) Scheme and corresponding images of a 3D printed cuboid with the encoded gridded globe as well as a boat‐shaped testing structure “benchy” with an anchor programmed into its hull are depicted. The same structures are shown after thermal treatment (80°C) resulting in the deletion of the information (scale bars = 5 mm).

The next step was the selection of a suitable photoinitiator (PI), which must remain colorless after polymerization to preserve high contrast of the programmed information. Additionally, it must enable efficient DLP printing of complex structures with low irradiation energy to avoid unwanted isomerization of Sp to Mc. Diphenyl(2,4,6‐trimethylbenzoyl)phosphine oxide (TPO) was selected as a suitable PI. Another important component of the ink formulation is monomers and crosslinkers, which define the final 3D polymer matrix. To preserve the information encoded in the structures, the locally controlled isomerized Sp or Mc must remain stable over time, requiring optimization of the matrix composition. Low‐polarity acrylates were chosen to minimize undesired isomerization while maintaining efficient fabrication of complex 3D structures [33]. As a starting point, the monomers isobornyl acrylate (IsobA; 55.0 wt%) and hexyl acrylate (HexA; 1.8 wt%) as well as the crosslinker tricyclo[5.2.1.0^2,6^]decanedimethanol diacrylate (TcddA; 40.0 wt%) (Figure 2a) were used to prepare the first ink formulation in combination with the PI TPO (3.0 wt%) and the photochromic Sp‐dA (0.2 wt%).

To determine optimal printing conditions, the curing depth of the ink was evaluated using a Jacobs working curve (Figure S1) [34]. A layer height of 100 µm was achieved with an irradiation time of 6 s at 405 nm (2.2 mW cm^−2^), while avoiding excessive exposure that could trigger unwanted isomerization. As all tested ink formulations showed similar curing behavior, these parameters were applied throughout (Table S1, Figures S1, S2, and S3).

As a next step, optimal conditions for the photoisomerization step (Sp → Mc) at 365 nm were assessed and applied to tune the composition of the ink formulation to enable the long‐term storage of programmed information. Therefore, motorcycle patterns were encoded inside of transparent fabricated cuboids using the multi‐wavelength 3D DLP printer. As expected, it was found that the polarity of the polymer matrix influences the isomerization kinetics. To optimize this and therefore minimize unwanted isomerization, HexA was replaced with hydroxyethyl acrylate (HEA), resulting in improved stability of the programmed information (Table S2, Figures S6 and S7). Furthermore, the influence of the crosslinker content, particularly TcddA, and the Sp‐dA content as the photochromic compound were studied, to avoid the gradual loss of color over time due to back‐isomerization in the dark (Table S2, Figure S8, Figures S9, S10, and S11). The optimal result was obtained with samples printed from the ink formulation comprising Sp‐dA (0.2 wt%, 0.1 mol%), IsobA (55.0 wt%, 62.8 mol%), TcddA (40.0 wt%, 31.3 mol%), HEA (1.8 wt%, 3.7 mol%), and TPO (3.0 wt%, 2.1 mol%), which remained colorless, while the programmed information stayed intact without detectable degradation for 35 days at −18°C in the dark (Figure S12), demonstrating excellent long‐term stability. To further evaluate visible‐light‐induced Mc to Sp isomerization, a programmed sample was irradiated with a high‐intensity 520 nm LED (71 mW cm^−^^2^). Even after 10 min of irradiation, only partial isomerization was observed, and the programmed information remained clearly readable (Figure S13). These results confirm the high stability of the optimized ink formulation, which was therefore used in all subsequent experiments. To evaluate the printing resolution, a 15 × 15 × 1 mm^3^ cuboid specimen containing four arrays of inserted lines with varying widths and spacings was printed. The minimal resolvable distance between the lines was determined at 92 µm (Figure S14).

To further characterize the difference in color between the printed and the colored material containing the programmed information, UV/Vis absorption spectroscopy of crosslinked films was performed (Figure 2b). After irradiation with 365 nm wavelength light, the characteristic signal for the Mc at 587 nm was detected [26]. The signals below 420 nm can mostly be attributed to the PI TPO [35].

The capability to DLP print and to encode complex 3D geometries was demonstrated by fabricating a transparent cube containing a programmed and embedded gridded globe as well as a printed boat‐shaped test structure, called “benchy,” with an anchor programmed into its hull (Figure 2c). To quantitatively assess the spatial resolution of the encoding, line features with systematically varied separations were inscribed throughout the entire volume of a transparent 3D‐printed cuboid. The minimum resolvable feature spacing was determined to be 246 µm (Figure S15), demonstrating the high fidelity and precision achievable with this approach. Finally, the encoded information can be erased by thermal treatment at 80°C, which triggers isomerization of the colored Mc form back to the colorless Sp state.

### 2.2 pH‐Triggered Reversible Encryption

**FIGURE 3:**
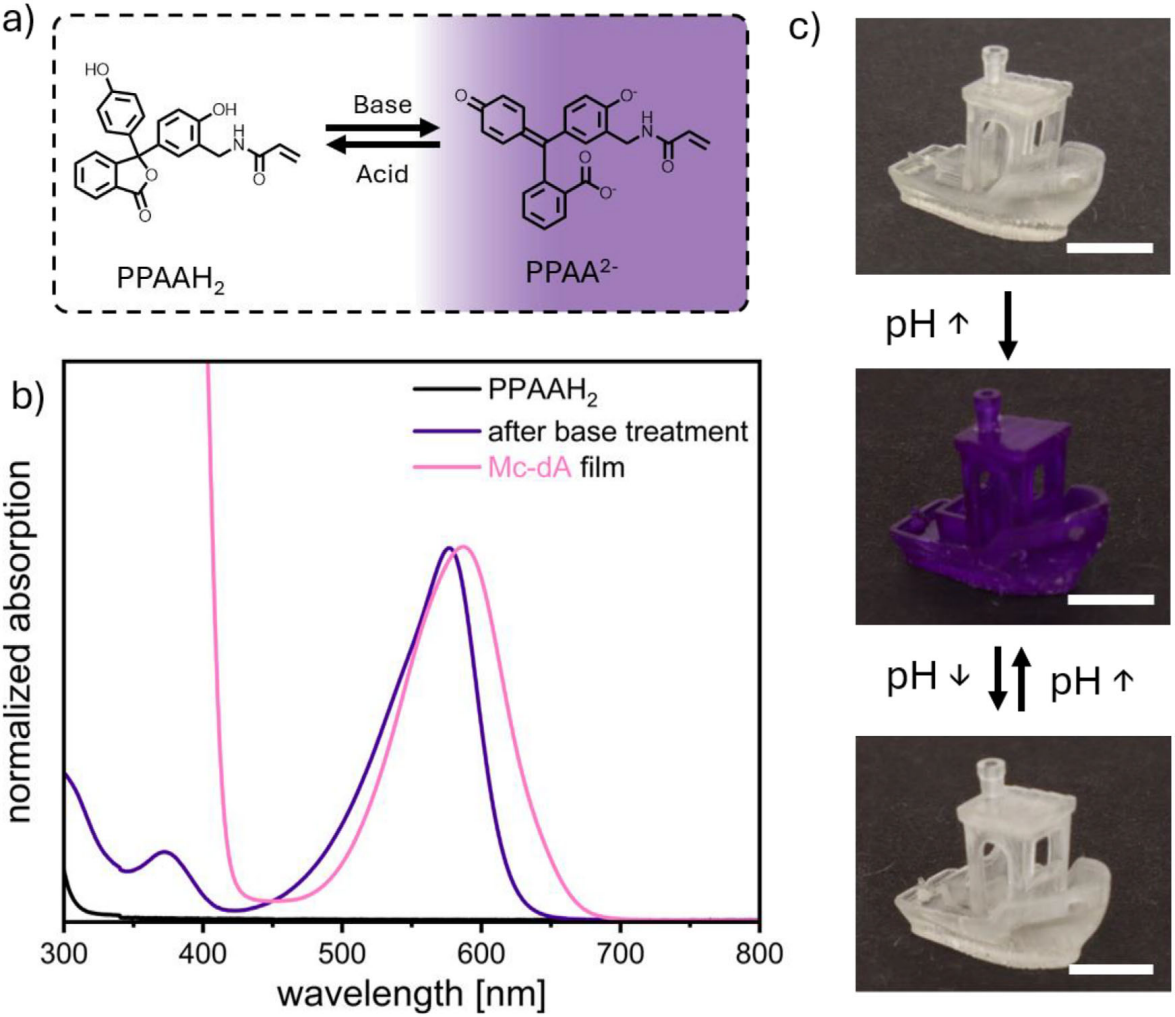
(a) pH‐responsive chromophore phenolphthalein acrylamide in the colorless (PPAAH_2_) and deprotonated colored (PPAA^−2^) form. (b) UV/Vis spectra of PPAAH_2_ (1.4 × 10^−4^ M) and PPAA^−2^ (1.8 × 10^−5^ M) dissolved in chloroform, as well as the UV/Vis spectrum of the Mc‐dA containing film are depicted. (c) Images of a 3D DLP printed “benchy” as printed (colorless), after immersing to a 0.5 M solution of TBD in acetone (purple), and after immersing the same structure again in a 0.5 M solution of AcOH in acetone (colorless) are shown (scale bars = 5 mm).

To enable reversible encryption of the programmable structures, a pH‐responsive chromophore compatible with the printable ink formulation was employed. The key selection criterion was an orthogonal stimulus‐induced color change from colorless to a strongly absorbing species with an absorption spectrum closely matching that of the merocyanine (Mc) within the polymer matrix, ensuring efficient masking of the encoded information. Phenolphthalein was identified as a suitable candidate, as it is colorless in its protonated form and develops a strong visible absorption upon deprotonation under basic conditions with a maximum at 577 nm [36], showing a significant spectral overlap with Mc (Figure 3a,b).

The pH‐responsive chromophore was incorporated into the 3D DLP printable ink formulation. For this purpose, phenolphthalein was functionalized with an acrylamide group (PPAAH_2_) to allow covalent integration into the polymer matrix (see SI, Section 3: Synthesis) [37, 38]. The modified chromophore was then added (1.0 wt%) to the previously optimized ink formulation in the absence of Sp‐dA to isolate and evaluate its color‐switching behavior. Therefore, the pH‐responsive ink formulation consisting of IsobA (54.5 wt%, 62.6 mol%), TcddA (39.7 wt%, 31.1 mol%), HEA (1.8 wt%, 3.7 mol%), TPO (3.0 wt%, 2.0 mol%), and PPAAH_2_ (1.0 wt%, 0.6 mol%) was employed to obtain a Jacobs working curve (Figure S4, Table S1). As expected, applying identical printing parameters (6 s, 405 nm wavelength LED, 2.2 mW cm^−2^) as used for the previous ink formulation resulted in comparable printability, enabling high‐resolution 3D fabrication.

To evaluate the pH‐triggered response, a boat‐shaped test structure, called “benchy,” was 3D printed and subsequently exposed to different pH environments. Immersion in an aqueous NaOH solution (0.5 M) did not induce any color change, likely due to limited diffusion of the base into the hydrophobic polymer matrix. In contrast, organic bases in acetone enabled activation of the chromophore. While DBU (0.5 M) induced only a slow and weak response, TBD (0.5 M) triggered a rapid and pronounced color change of the initially colorless “benchy” to a deep purple within < 60 s, corresponding to the conversion of PPAAH_2_ to PPAA^−2^ (Figure 3a,c). Lower TBD concentrations (0.3 and 0.1 M) also induced the response, albeit more slowly and with reduced intensity. Therefore, 0.5 M TBD in acetone was identified as the optimal encryption conditions. Restoration of the colorless material was achieved by immersing the purple “benchy” in 0.5 M acetic acid in acetone for 30 s, to recover the PPAAH_2_ form (Figure 3c and Figure S16). This process could be repeated over five cycles without any observable structural degradation or loss in color intensity, underscoring the reversibility of the system. The color‐switching behavior was further corroborated by UV/Vis spectroscopy using a crosslinked film of the material (Figure S17). Prior to treatment with 0.5 M TBD in acetone, no absorption was observed above 420 nm and after exposure to TBD, a new absorption band at 584 nm was detected. These results demonstrate that PPAA^−2^ effectively masks the Mc absorption band, highlighting its potential for information encryption.

### 2.3 Orthogonal, Reversible Encryption of Encoded 3D Structures

**FIGURE 4:**
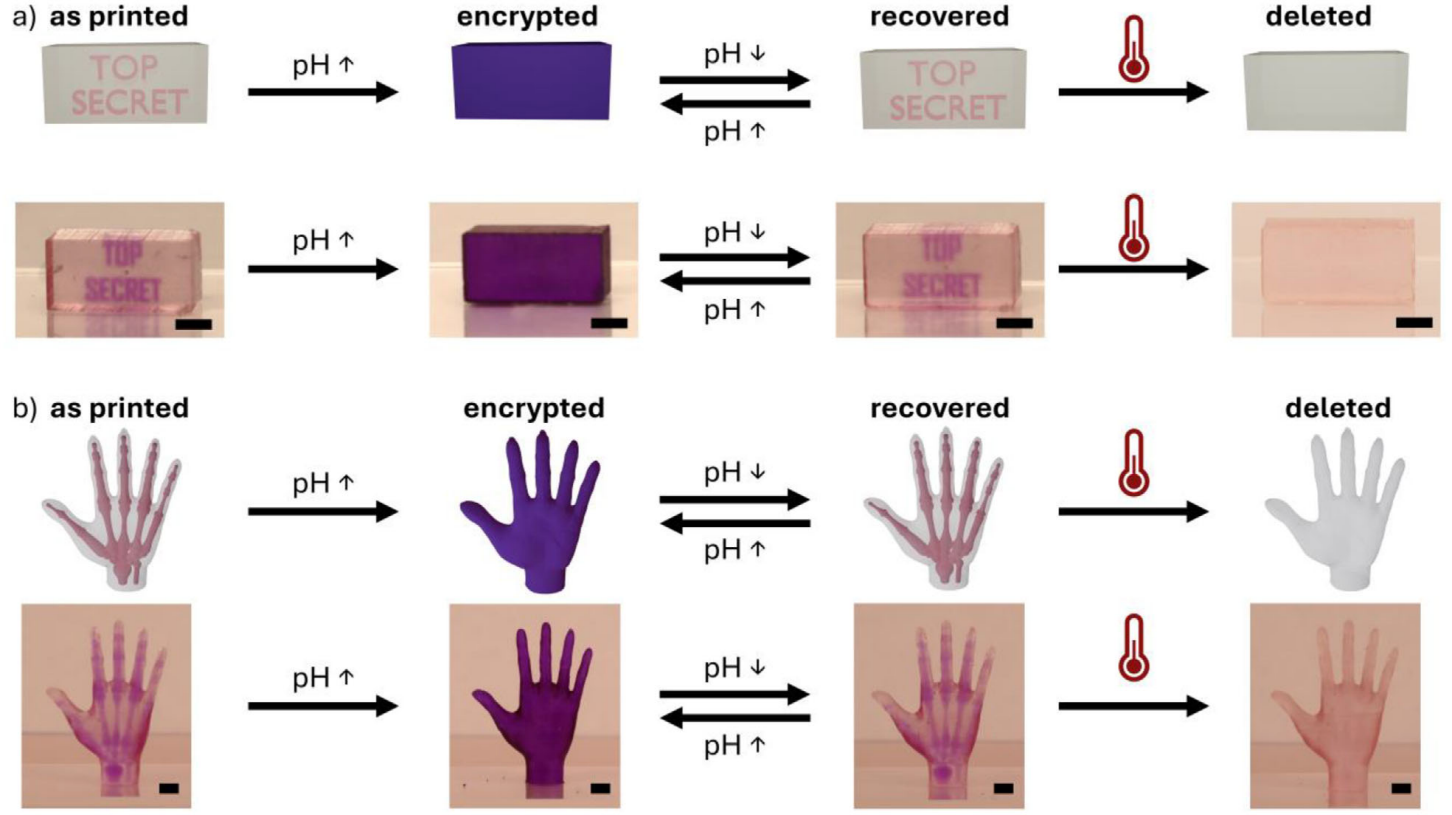
(a) Scheme and corresponding images of a 3D printed cuboid with the encoded message “TOP SECRET” as‐printed, after encryption in basic media, recovered, and deleted after thermal treatment. (b) Scheme and images of a 3D‐printed hand with embedded bone features, displayed across the same states (scale bars = 2 mm).

To realize encoding in 3D structures with the capability to reversibly encrypt the information, 0.2 wt% Sp‐dA and PPAAH_2_ were combined in the ink formulation. Using established printing and encoding conditions, cuboid specimens with encoded lettering (“top secret”) were fabricated, yielding initially colorless structures with clearly visible information. To achieve complete encryption, the PPAAH_2_ content was optimized. While ink formulations containing 1.0 and 3.0 wt% PPAAH_2_ resulted in only partial masking upon treatment with 0.5 M TBD in acetone (Figure S18, Table S3), a content of 5.0 wt% PPAAH_2_ in the ink formulation enabled full encryption within 30 s, rendering the encoded information undetectable (Figure 4a). Importantly, the encrypted state could be fully reversed by treatment with 0.5 M acetic acid in acetone, and the cycle was repeated at least five times without observable degradation or swelling (Figure S20). Permanent deletion of the information was achieved by thermal treatment at 80°C, inducing the conversion from Mc to Sp. The optical properties of a film containing both chromophores were also investigated via UV/Vis spectroscopy. While no absorption at wavelengths longer than 415 nm was observed when Sp and PPAAH_2_ are present in the material, the isomerization of Sp to Mc resulted in a new absorption maximum at 580 nm. Also, a new absorption maximum at 582 nm was detected when only PPAAH_2_ was isomerized to PPAA^−2^ (Figure S18). The high similarity of the absorption spectra of the films containing Mc or PPAA^−2^ further highlights the high fidelity of the encryption.

Finally, the system was extended to more complex architectures to demonstrate its capability for 3D information encoding and encryption. To this end, a hand‐shaped structure with internally programmed bone features was fabricated via multi‐wavelength 3D DLP printing (Figure 4b). Upon immersion in a basic solution, the embedded bone pattern became encrypted, accompanied by a uniform transition of the hand to a deep purple color. Decryption was readily achieved by immersing the structure in an acetic acid solution in acetone for 30 s, restoring the colorless state and rendering the internal bone features visible once again. This reversible encryption–decryption cycle was repeated five times without any detectable loss in structural integrity or optical performance, underscoring the durability of the system. Complete and permanent erasure of the encoded information can be accomplished by thermal treatment at 80°C, yielding the original 3D shape without encoded information.

## 3 Conclusion

In summary, we have successfully developed an ink formulation containing a photochromic Sp‐dA that enabled the fabrication of programmable 3D structures via dual‐wavelength DLP printing. Using a 405 nm wavelength LED for printing and a 365 nm wavelength LED for programming, information was encoded with high spatial control in 3D through localized isomerization from the colorless Sp to the colorful Mc form. It was found that the polymeric matrix played a crucial role. The optimized system exhibited excellent stability with no detectable degradation or bleaching of the printed structures or encoded information after storage for up to 35 days in the dark at −18°C. The programmed information can be deleted on demand, upon heating to 80°C, which induces the isomerization of the Mc to the Sp in the polymer matrix. Furthermore, reversible encryption was introduced as an orthogonal functionality by incorporating the pH‐responsive chromophore PPAAH_2_, which undergoes a pronounced and reversible color change under basic conditions. By integrating both photo‐ and pH‐responsive components into a single ink formulation, complex 3D structures with embedded information are realized that can be selectively encrypted or decrypted on demand. Our study introduces the novel combination of smart materials to obtain a multi‐responsive system triggered by orthogonal stimuli. We believe that this multi‐responsive system is a promising candidate for advanced information storage, security, and sensing applications. This work provides a foundation for future developments in multi‐responsive 3D materials with programmable and adaptive functionalities.

## Author Contributions

The manuscript was written through contributions of all authors. All authors have given approval to the final version of the manuscript.

## Conflicts of Interest

The authors declare no conflicts of interest.

## Supporting information




**Supporting File 1**: anie73606‐sup‐0001‐SuppMat.pdf.

## Data Availability

The data that support the findings of this study are openly available in HeiDATA at https://doi.org/10.11588/DATA/6WDCCM.
