# Supplementary material for: Encoding and Encryption via Dual‐Wavelength 3D Digital Light Processing
Source: Angew Chem Int Ed Engl. 2026 Jul 17;65(38):e1978756. doi: 10.1002/anie.1978756 (PMC13573139; doi:10.1002/anie.1978756)
Supplement: Supplementary file 1 [file ANIE-65-e1978756-s001.pdf]

Supporting Information

Encoding and Encryption via Dual-Wavelength 3D Digital Light Processing

Finn Kröger, *Michal P. Swiatek*, and *Eva Blasco**

Outline:

1. Materials 2
2. Methods 2
3. Synthesis 4
4. Jacobs Working Curves 5
5. Ink Formulation Optimization for Enhanced Information Stability 8
6. Resolution Tests 12
7. Sectioning pH-Responsive Colored Sample 13
8. UV/Vis Spectra 13
9. Optimization of the Final Ink Formulation 15
10. Repeatable pH-Response 15
11. ^{1}H NMR Spectra 16
12. References 17

1. Materials

Isobornyl acrylate (IsobA), tricyclo[5.2.1.0^{2,6}]decanedimethanol diacrylate (TcddA), hydroxyethyl acrylate (HEA), hexyl arylate (HexA), and diphenyl(2,4,6-trimethylbenzoyl)phosphine oxide (TPO) were bought from Sigma Aldrich GmbH. Deuterated solvents were purchased from Deutero GmbH. All other employed chemicals and solvents were purchased from Sigma Aldrich GmbH, Fisher Scientific, BLDpharm or TCI. All reagents were used without further purification.

2. Methods

All experiments were performed under yellow light conditions.

Ink Formulation preparation: For all ink formulations, the non-responsive components were combined and sonicated at room temperature for 10 min to obtain the stock ink formulation. The responsive compound (Sp-dA or $PPAAH_2$) was afterwards combined with the stock ink formulation and the mixture was shaken for 30 min before it was used for DLP printing. The stock solution of the final formulation consisted out of IsobA (2614 mg, 52.3 wt%), TcddA (1901 mg, 38.0 wt%), HEA (86 mg, 1.7 wt%), and TPO (142 mg, 2.8 wt%) and was sonicated for 10 min at room temperature. Afterwards, $PPAAH_2$ (250 mg, 5.0 wt%) and Sp-dA (10 mg, 0.2 wt%) were added to the stock solution, which was then shaken for 30 min before it was used for DLP printing. All ink formulations were stable for several weeks when stored in the dark, and shaken for 30 min before they were used again.

Curing tests: The curing test was performed on the DLP printer with a 405 nm wavelength LED at an intensity of 2.2 mW cm^{-2}. A drop of the respective ink formulation was applied to a glass coverslip (Marienfeld, 170 ± 5 µm) and irradiated with a circle (diameter 2 mm) for a certain time. The uncured resin was washed away with acetone and the spot was carefully dried applying pressurized nitrogen. The thickness of the cured material was measured with a caliper. The exposure energy was semi logarithmically plotted against the curing depth to obtain the Jacobs working curve.[1]

Digital light processing (DLP): For multi-wavelength 3D DLP printing a commercial DLP printer called MONO3Z4 (Microfluidics for all, inc, USA) equipped with two LEDs was applied. The 405 nm wavelength LED (2.2 mW cm^{-2}) was used to initiate the polymerization of the ink formulation to print 3D structures. The 365 nm wavelength LED (2.8 mW cm^{-2}) was applied to program the information into 3D printed structures by switching the Sp to Mc. A layer thickness of 100 µm was used for all prints and the software MonoWare (Microfluidics for all, inc., USA) was applied to slice the digital 3D models. After printing all structures were developed for 5 min in acetone and carefully dried with pressurized nitrogen. For all printed ink formulations irradiation times of 6 s (405 nm LED 2.2 mW cm^{-2}) were used for printing and irradiation times of 15 s (365 nm LED 2.8) were used for the encoding.

UV/Vis spectroscopy: A Jasco V-770 Spectrophotometer (Jasco Deutschland GmbH) was used to record all UV/Vis spectra. To measure the spectrum of $PPAAH_2$ (1.4 x 10^{-4} M) in chloroform, a quartz glass cuvette (volume 3 mL) with a 1.0 cm diameter was used. 0.15 mL of a 0.5 M TBD solution in chloroform were added to the cuvette to obtain the $PPAA^{-2}$ (1.8x 10^{-5} M) for the respective spectrum. The film holder FIH-741 was applied to record all UV/Vis spectra of films. The respective formulation was cured by applying a 365 nm wavelength LED (6.7 mW cm^{-2}) for 2 min. To obtain the Sp-dA in a film it was heated to 80 °C. To obtain the $PPAA^{-2}$ in a film, it was immersed into a 0.5 M solution of TBD in acetone for 1 min and rinsed with acetone before the spectrum was recorded.

NMR spectroscopy: All NMR spectra were recorded on a *Bruker Avance III 300 MHz* or *Bruker Avance III 500 MHz* and processed with MestReNova 14.3.2-32681. Chemical shifts are given in parts per million. The references for the ^{1}H NMR spectra were referenced to the locked solvents. The signal patterns are noted as follows: s (singlet), d (doublet), t (triplet), dd (doublet of a doublet), m (multiplet).

RGB value determination: The values were extracted from the respective images using ImageJ and averaged over more than 3600 pixels.

3. Synthesis

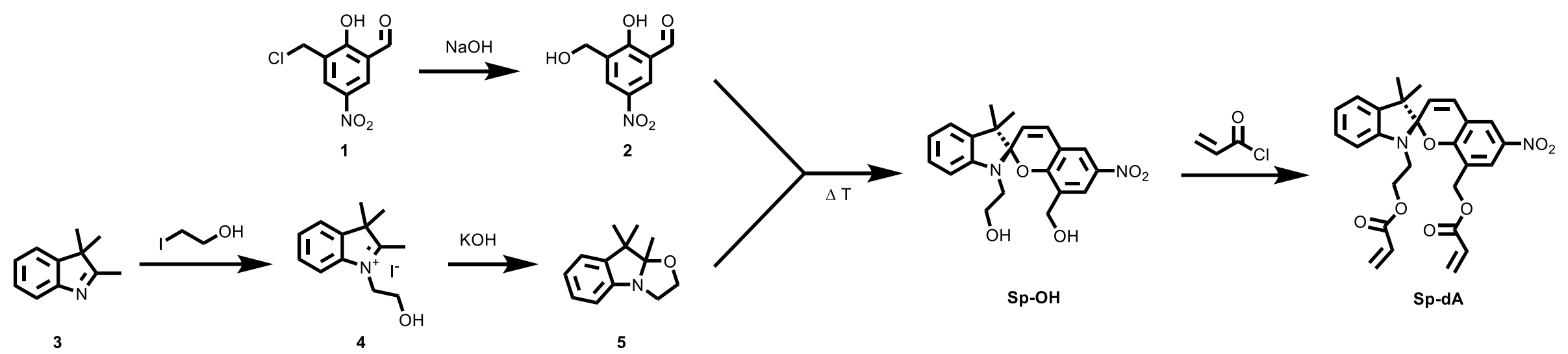

Scheme S1: Overview of the synthesis route for Sp-dA.

To obtain the Sp-dA a literature known synthesis rout was performed.[2] A hydrolysis was performed with compound **1** applying NaOH to obtain **2**. Compound **3** was converted with 2-iodoethanol to yield compound **4**, which was further converted using KOH to obtain compound **5**. Under a nitrogen atmosphere in absolute ethanol, the Fischer condensation applying **2** and **5** was performed to obtain **Sp-OH**. The polymerizable moieties were introduced via an esterification and Sp-dA was obtained as a dark red solid. ^{1}H NMR (500 MHz, acetone-d_6) δ = 8.1 (s, 2H), 7.2 (d, *J* = 10.4 Hz, 1H), 7.2 – 7.12 (m, 2H), 6.8 (t, *J* = 7.4 Hz, 1H), 6.8 (d, *J* = 7.7 Hz, 1H), 6.3 – 6.3 (m, 2H), 6.1 – 6.1 (m, 3H), 5.9 – 5.8 (m, 2H), 5.0 (s, 2H), 4.4 – 4.3 (m, 2H), 3.7 – 3.6 (m, 1H), 3.6 – 3.50 (m, 1H), 1.3 (s, 3H), 1.2 (s, 3H) ppm.

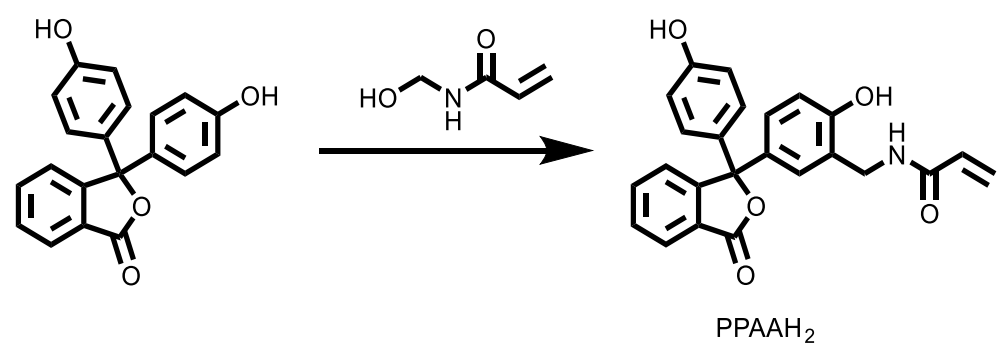

Scheme S2: Synthesis of the PPAAH$_2$.

According to literature,[3,4] in a round bottom flask at 0°C, N-(hydroxymethyl)acrylamide (978 mg, 9.67 mmol), aluminum chloride (1.29 g, 9.67 mmol), and phenothiazine (1 mg) were dissolved in 4 mL acetone. Following, the reaction mixture was heated to 40 °C and phenolphthalein (3.98 g, 12.5 mmol) dissolved in 8 mL acetone was added over the course of 5 min. The reaction was stirred at 40 °C for 16 h before 100 mL water was added. The aq. phase was extracted with dichloromethane (3 x 50 mL) and the org. phase was dried over $MgSO_4$. The crude product was purified via column chromatography (SiO_2, EtOAc: dichloromethane 1:1). The pure product was obtained as a colorless crystalline powder (28%, 1.10 g, 2.74 mmol). ^{1}H NMR (301 MHz, DMSO-d_6) δ = 9.9 (s, 1H), 9.6 (s, 1H), 8.5 – 8.4 (m, 1H), 7.9 (d, *J* = 7.6 Hz, 1H), 7.8 – 7.7 (m, 1H), 7.7 – 7.6 (m, 2H), 7.1 – 7.0 (m, 5H), 6.8 (d, *J* = 8.4 Hz, 1H), 6.8 – 6.7 (m, 2H), 6.3 – 6.2 (m, 1H), 6.1 (dd, *J* = 17.1, 2.4 Hz, 1H), 5.6 (dd, *J* = 10.0, 2.4 Hz, 1H), 4.2 (d, *J* = 5.8 Hz, 2H) ppm.

4. Jacobs Working Curves

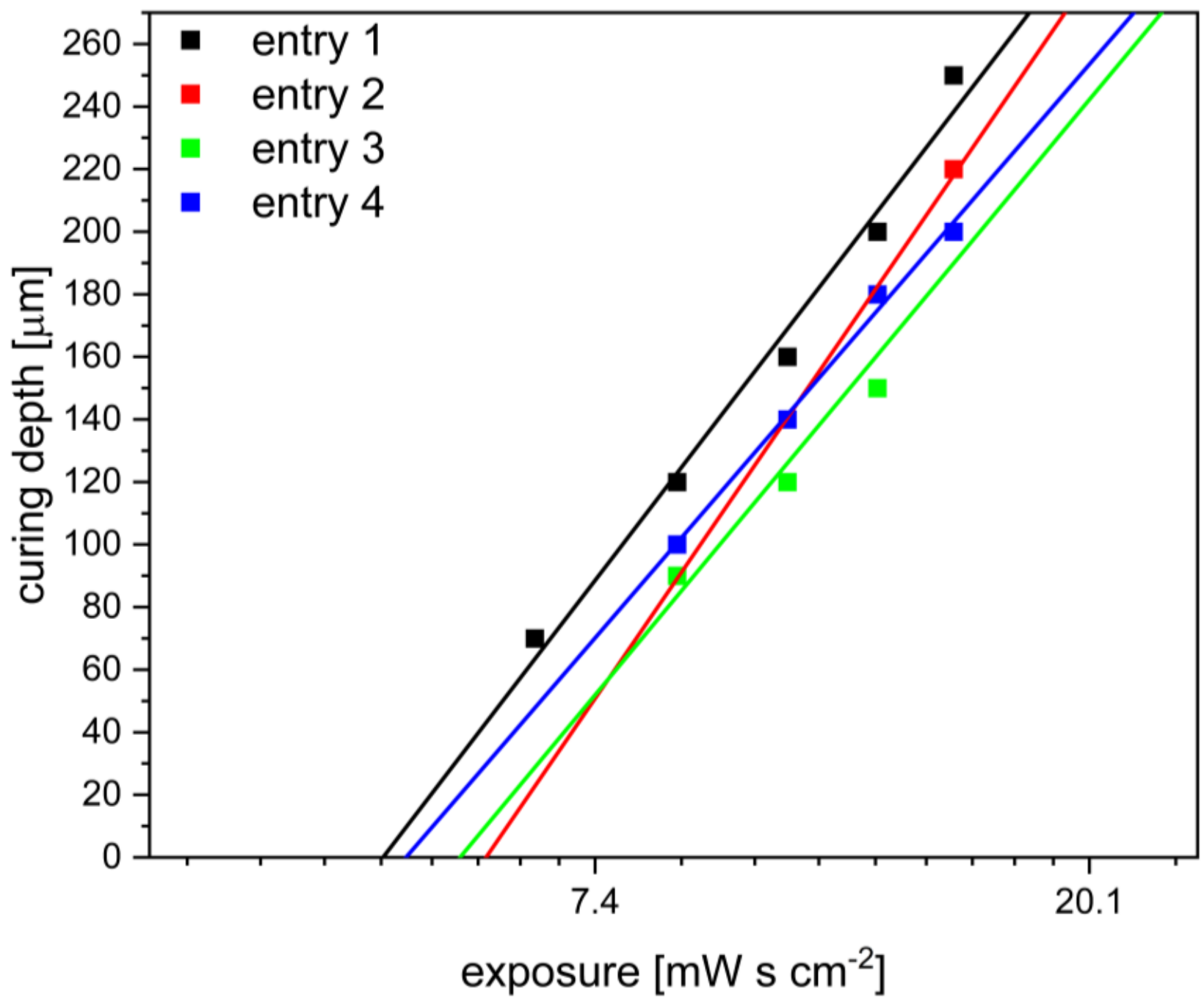

Figure S1: Jacobs working curve of the ink formulations listed in **Table S2** under entry 1, 2, 3, and 4 applying the 405 nm wavelength LED of the DLP printer.

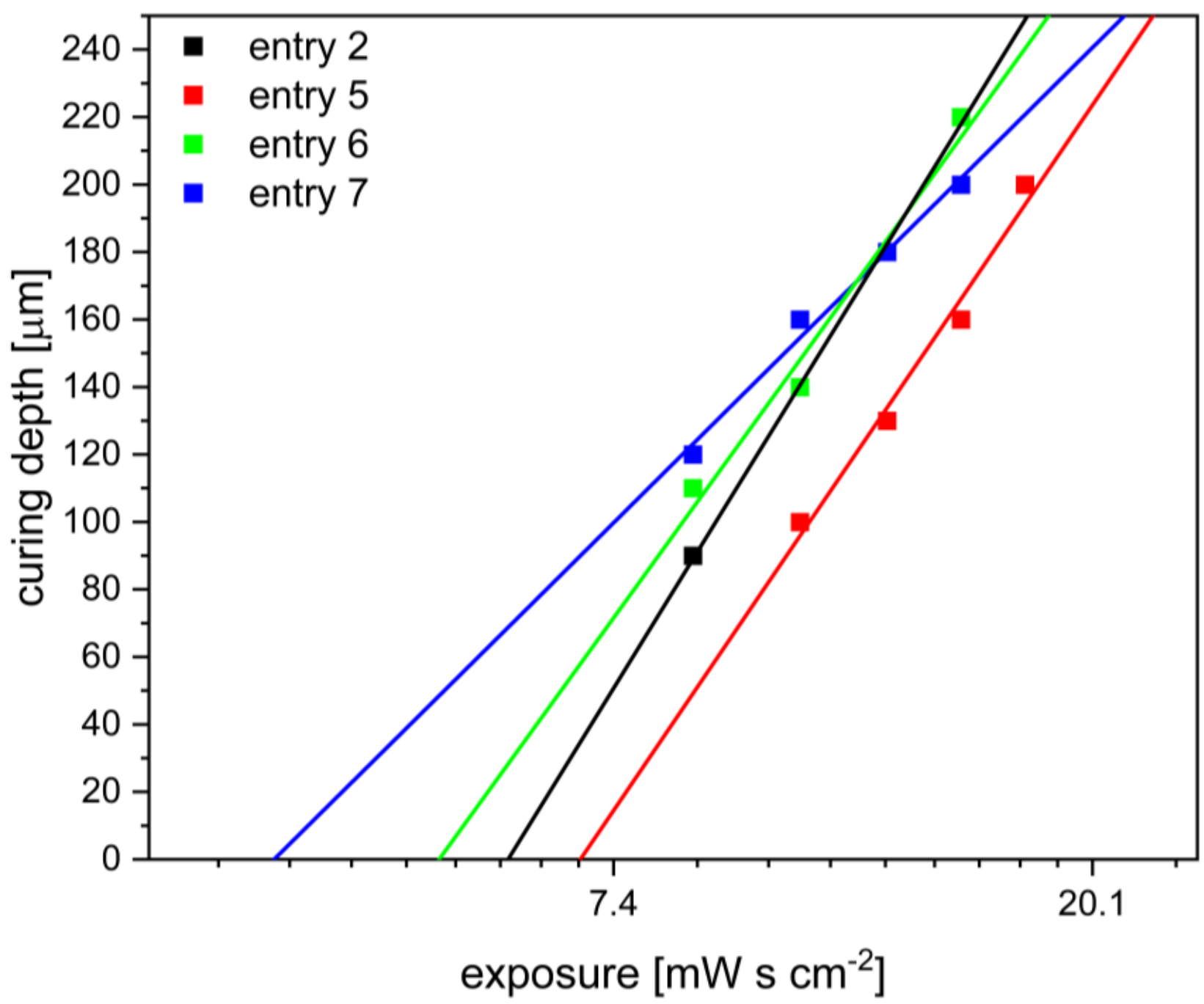

Figure S2: Jacobs working curve of the ink formulations listed in **Table S2** under Entry 2, 5, 6, and 7 applying the 405 nm wavelength LED of the DLP printer.

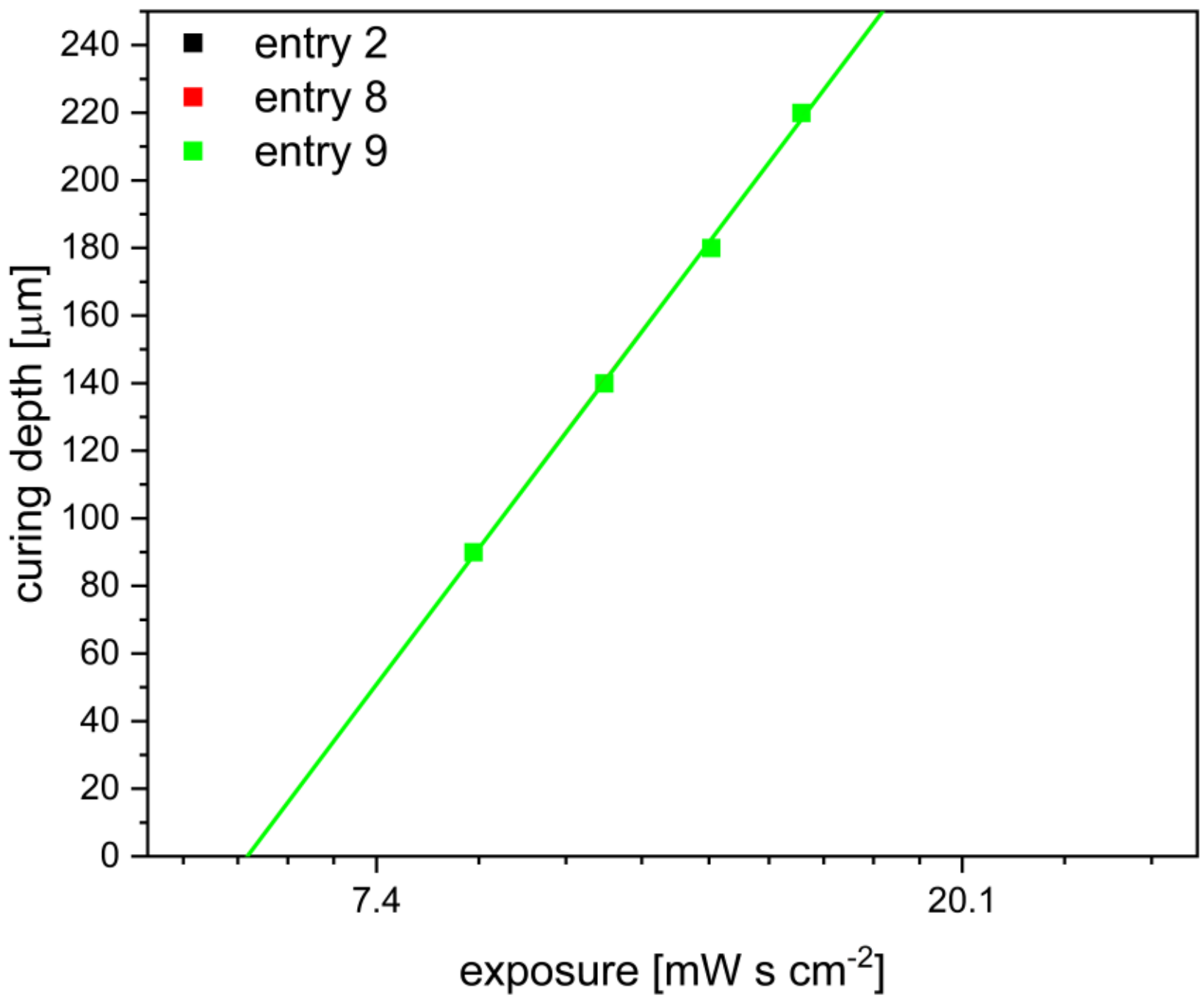

Figure S3: Jacobs working curve of the ink formulations listed in **Table S2** under entry 2, 8, and 9 applying the 405 nm wavelength LED of the DLP printer. The three graphs cover each other.

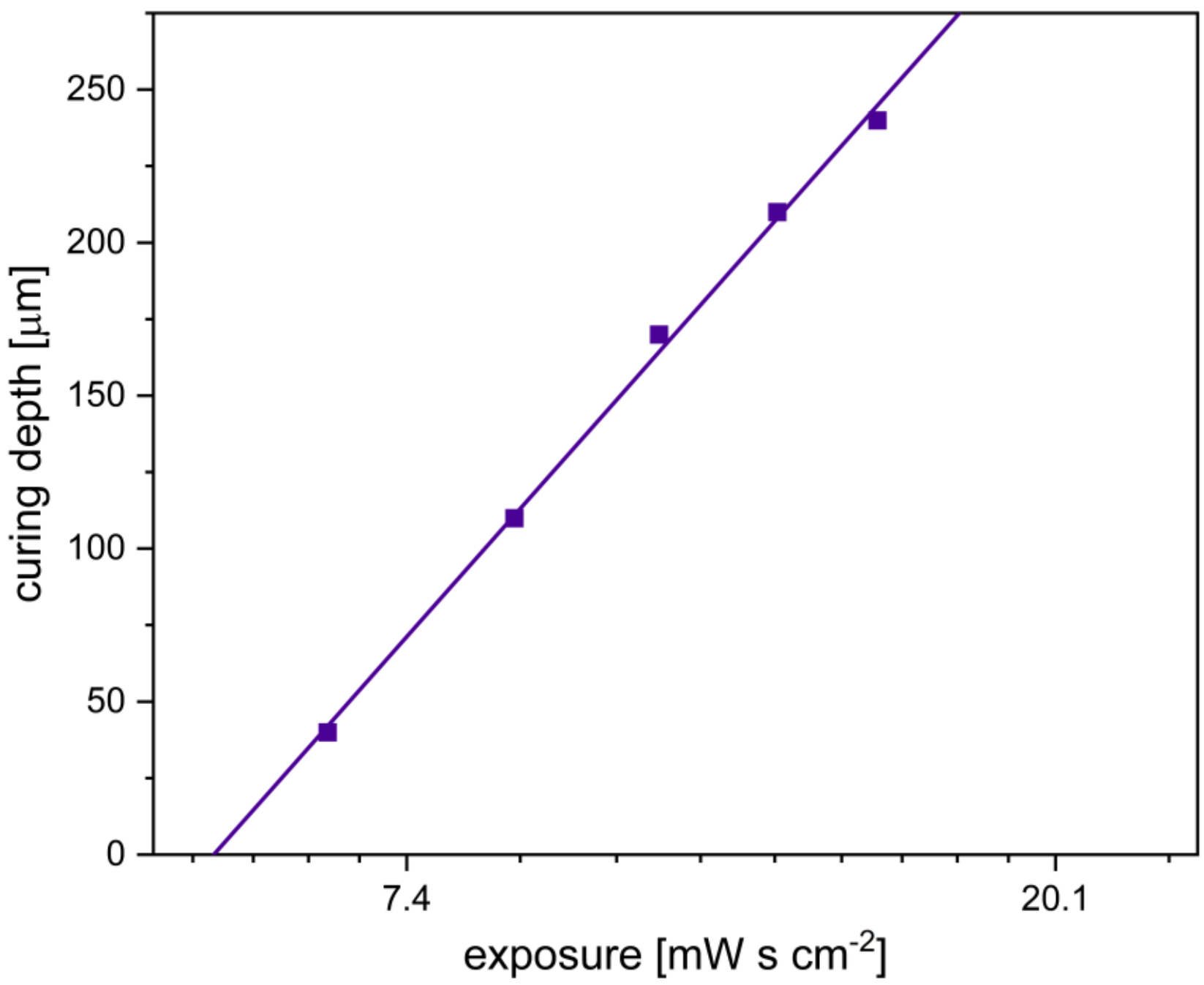

Figure S4: Jacobs working curve of the ink formulation containing the chromophore $PPAAH_2$ applying the 405 nm wavelength LED of the DLP printer.

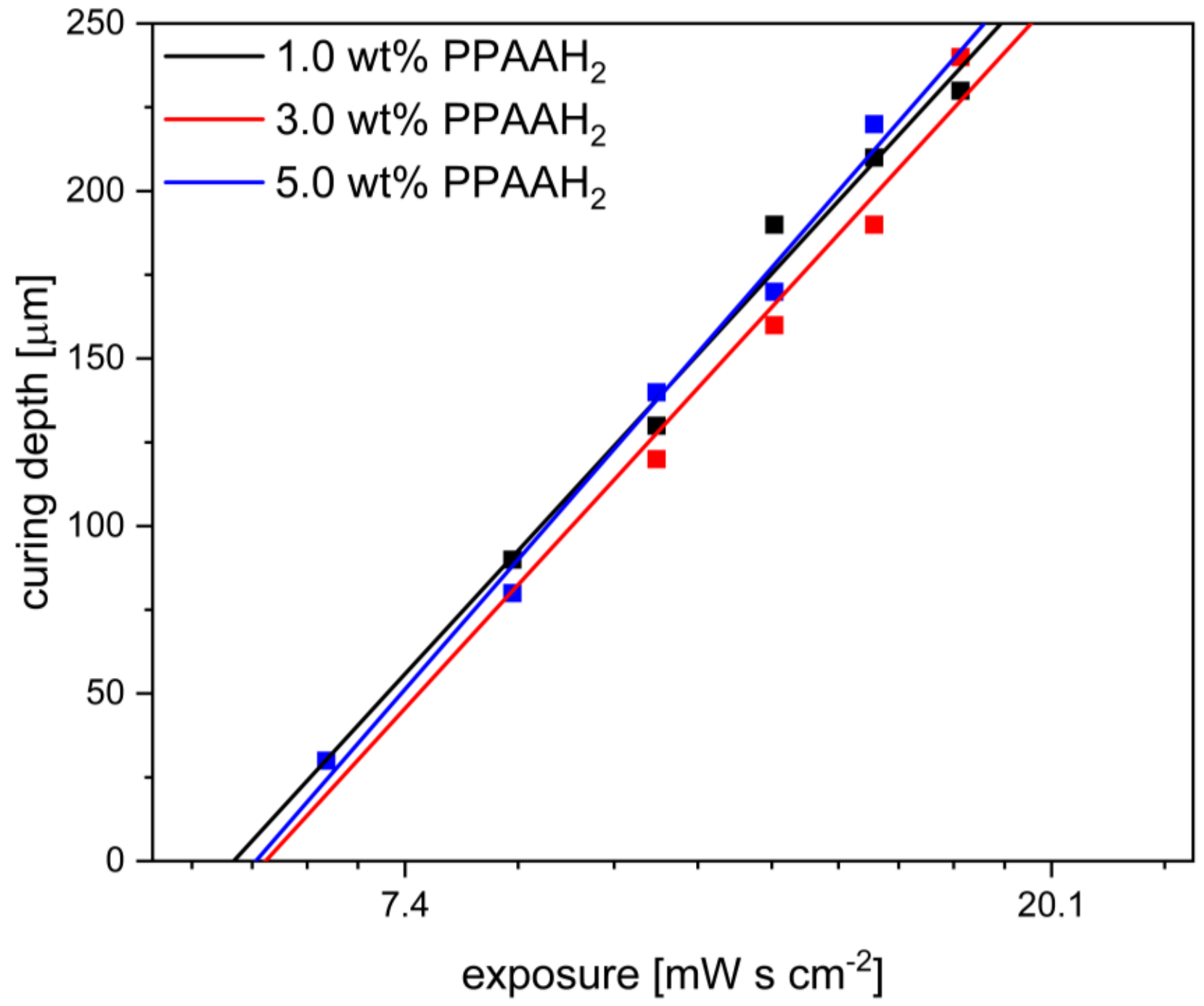

Figure S5: Jacobs working curve of the ink formulations, containing 0.2 wt% Sp-dA and 1.0, 3.0, or 5.0 wt% $PPAAH_2$ respectively applying the 405 nm wavelength LED of the DLP printer.

In a Jacobs working curve, the interception with the X-axis describes the necessary exposure energy to reach the gel point of the ink formulation, which is termed critical energy (E_c). The penetration depth (D_p) of the ink formulation describes the point till the intensity of the light is reduced to e^{-1} (~37%) of the initial intensity and is given by the slope of the Jacobs working curve.[1]

Table S1: The determined E_c and D_p of the respective Jacobs working curve are listed.

ink formulation / Jacobs working curve	E_c [mJ cm^{-2}]	D_p [µm]
**Figure S1** entry 1	4.8	206
**Figure S1** entry 2	6.0	230
**Figure S1** entry 3	5.6	190
**Figure S1** entry 4	5.1	183
**Figure S2** entry 5	6.9	209
**Figure S2** entry 6	5.2	196
**Figure S2** entry 7	3.7	141
**Figure S3** entry 8	6.0	230
**Figure S3** entry 9	6.0	230
**Figure S4**	5.5	239
**Figure S5** 1.0 wt% $PPAAH_2$	5.7	211
**Figure S5** 1.0 wt% $PPAAH_2$	6.0	211
**Figure S5** 1.0 wt% $PPAAH_2$	5.9	222

5. Ink Formulation Optimization for Enhanced Information Stability

Table S2: Tested ink formulations, to reduce unwanted isomerization between the Sp and Mc form in the 3D printed structures. The components investigated are highlighted for the respective entries.

entry	Sp-dA	IsboA	TcddA	HEA	HexA	TPO
1	0.2 wt%	55.0 wt%	40.0 wt%	-	1.8 wt%	3.0 wt%
2	0.2 wt%	55.0 wt%	40.0 wt%	1.8 wt%	-	3.0 wt%
3	0.2 wt%	51.6 wt%	40.0 wt%	5.2 wt%	-	3.0 wt%
4	0.2 wt%	42.8 wt%	40.0 wt%	15.0 wt%	-	3.0 wt%
5	0.2 wt%	75.0 wt%	20.0 wt%	1.8 wt%	-	3.0 wt%
6	0.2 wt%	45.0 wt%	50.0 wt%	1.8 wt%	-	3.0 wt%
7	0.2 wt%	35.0 wt%	60.0 wt%	1.8 wt%	-	3.0 wt%
8	0.1 wt%	55.1 wt%	40.0 wt%	1.8 wt%	-	3.0 wt%
9	0.4 wt%	54.9 wt%	39.9 wt%	1.8 wt%	-	3.0 wt%
Optimized	0.2 wt%	55.0 wt%	40.0 wt%	1.8 wt%	-	3.0 wt%

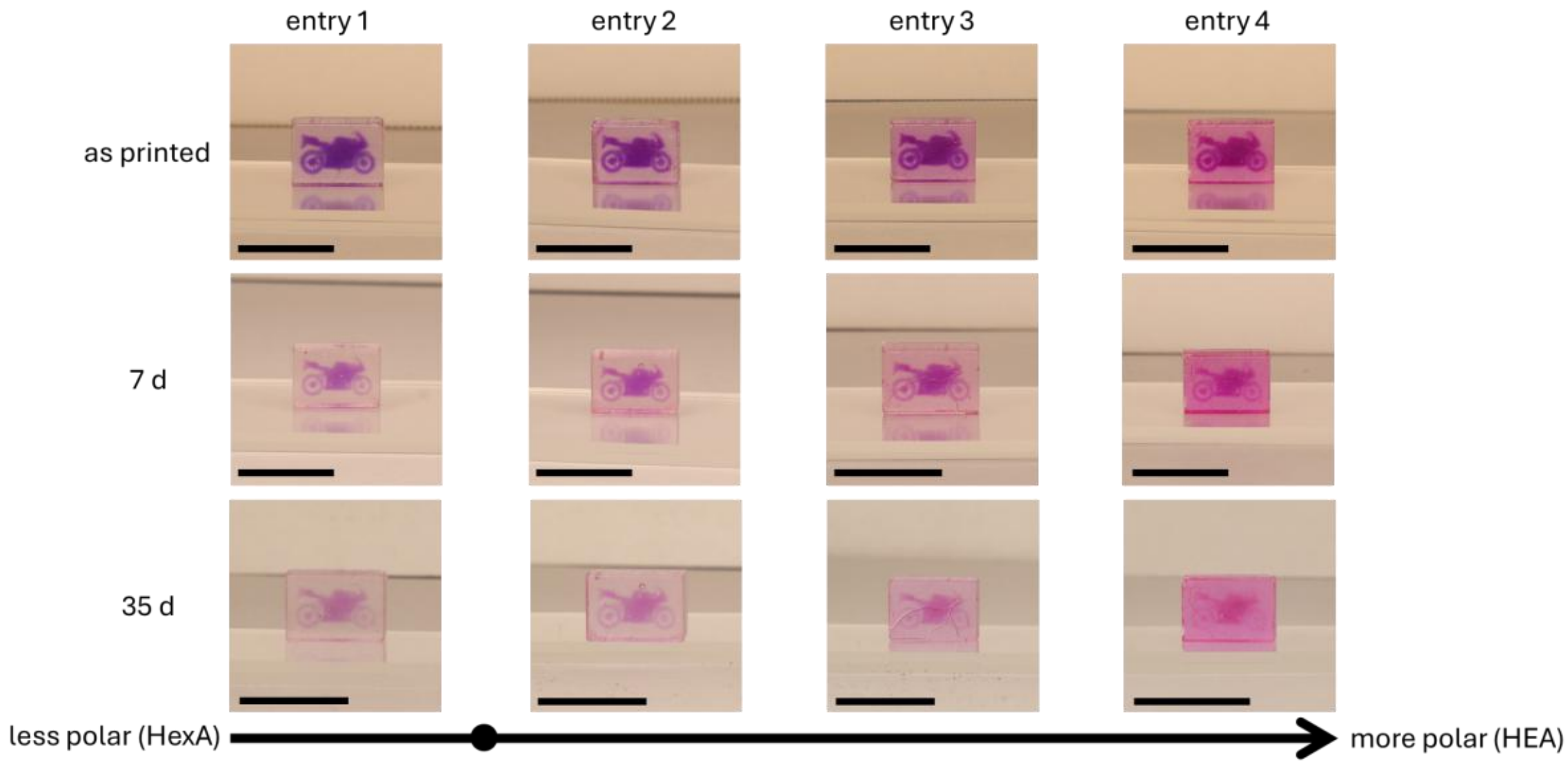

Figure S6: Images of programmed structures printed from ink formulations listed under entry 1,2,3, and 4 in **Table S2** are shown directly after the printing, after 7 days and after 35 days. The samples were stored in the dark at 20 °C (scale bars = 1 cm).

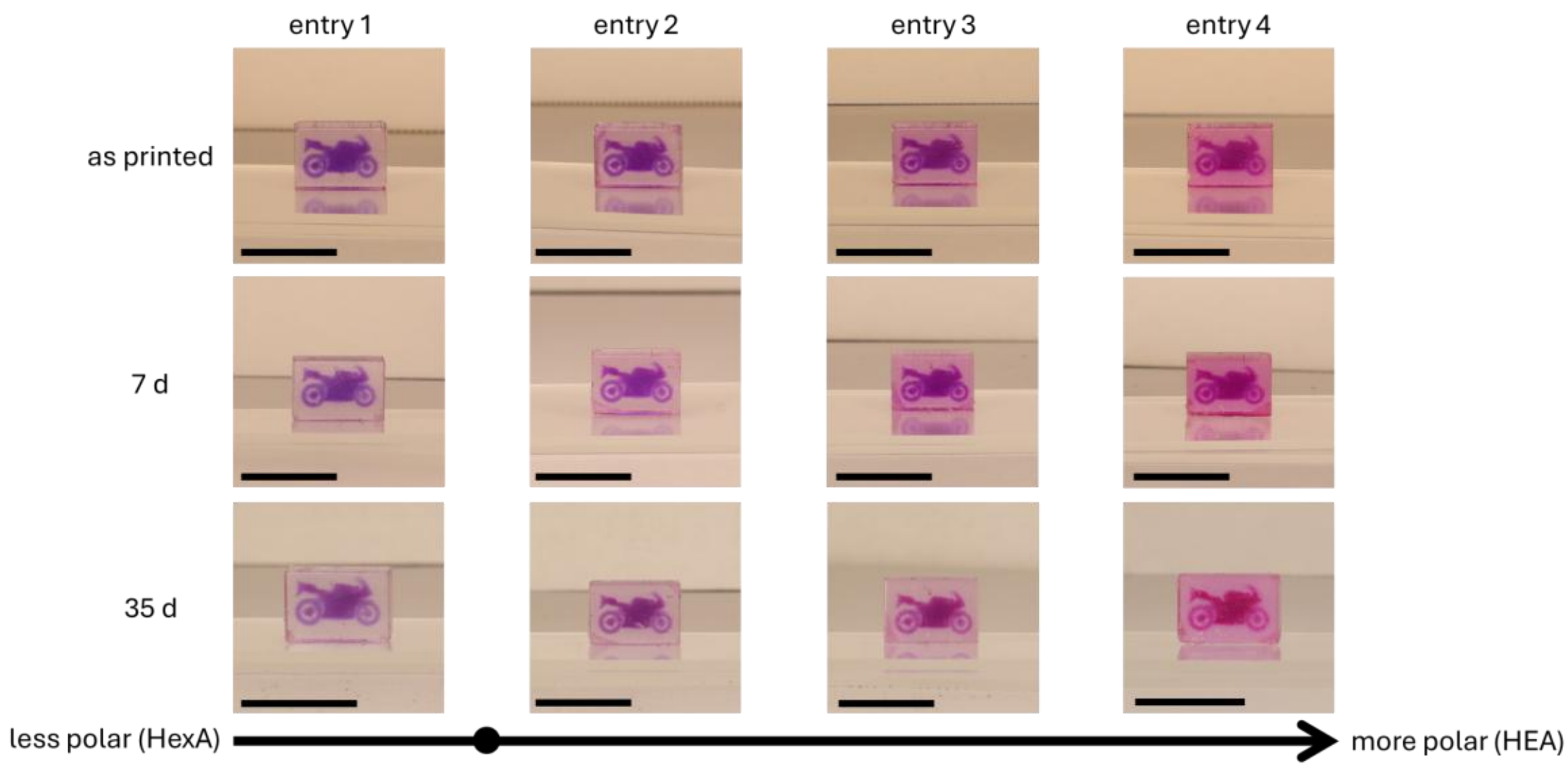

Figure S7: Images of programmed structures printed from ink formulations listed under entry 1,2,3, and 4 in **Table S2** are shown directly after the printing, after 7 days and after 35 days. The samples were stored in the dark at -18 °C (scale bars = 1 cm).

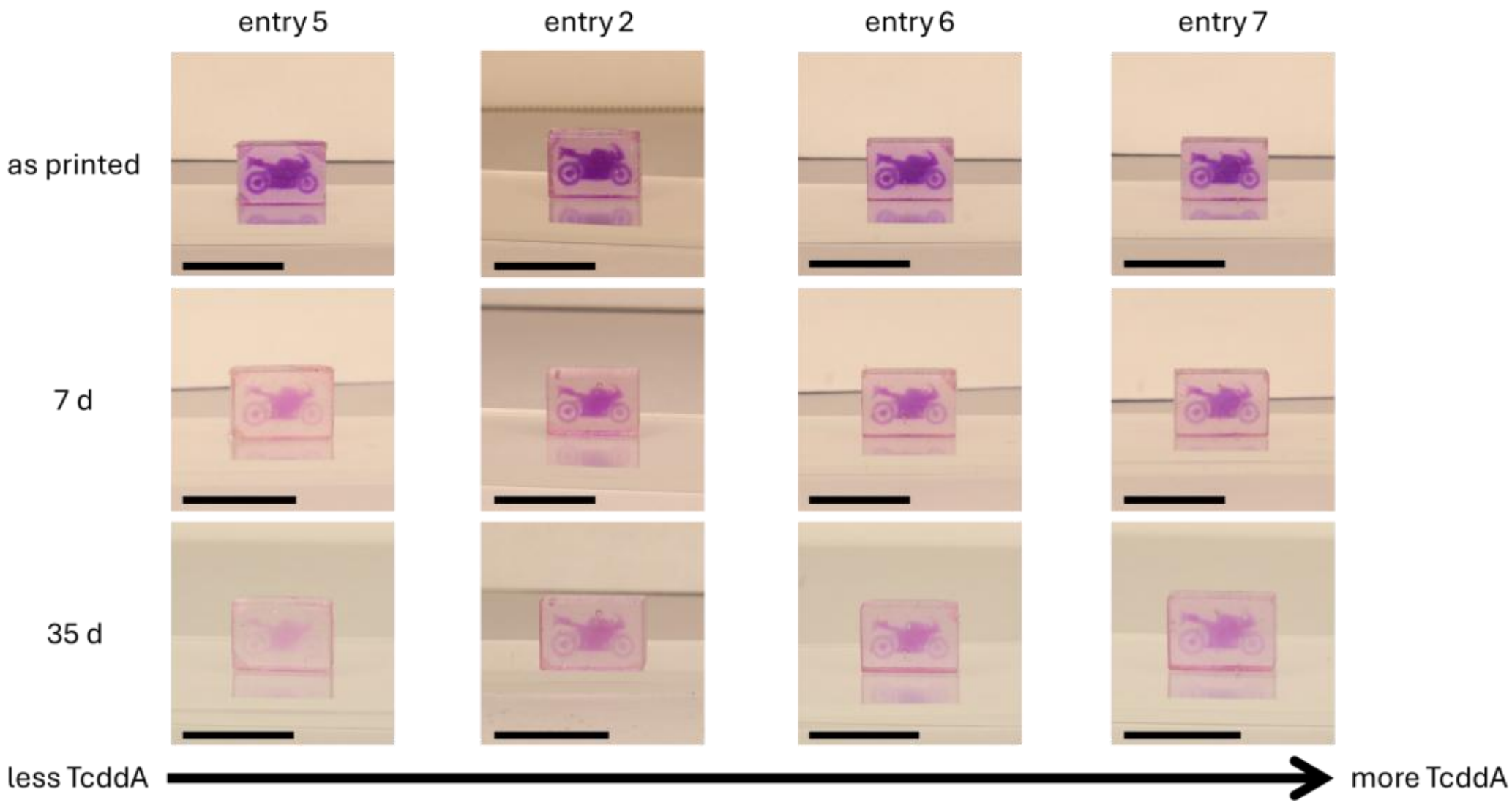

Figure S8: Images of programmed structures printed from ink formulations listed under entry 2, 5, 6 and 7 in **Table S2** are shown directly after the printing, after 7 days and after 35 days. The samples were stored in the dark at 20 °C (scale bars = 1 cm).

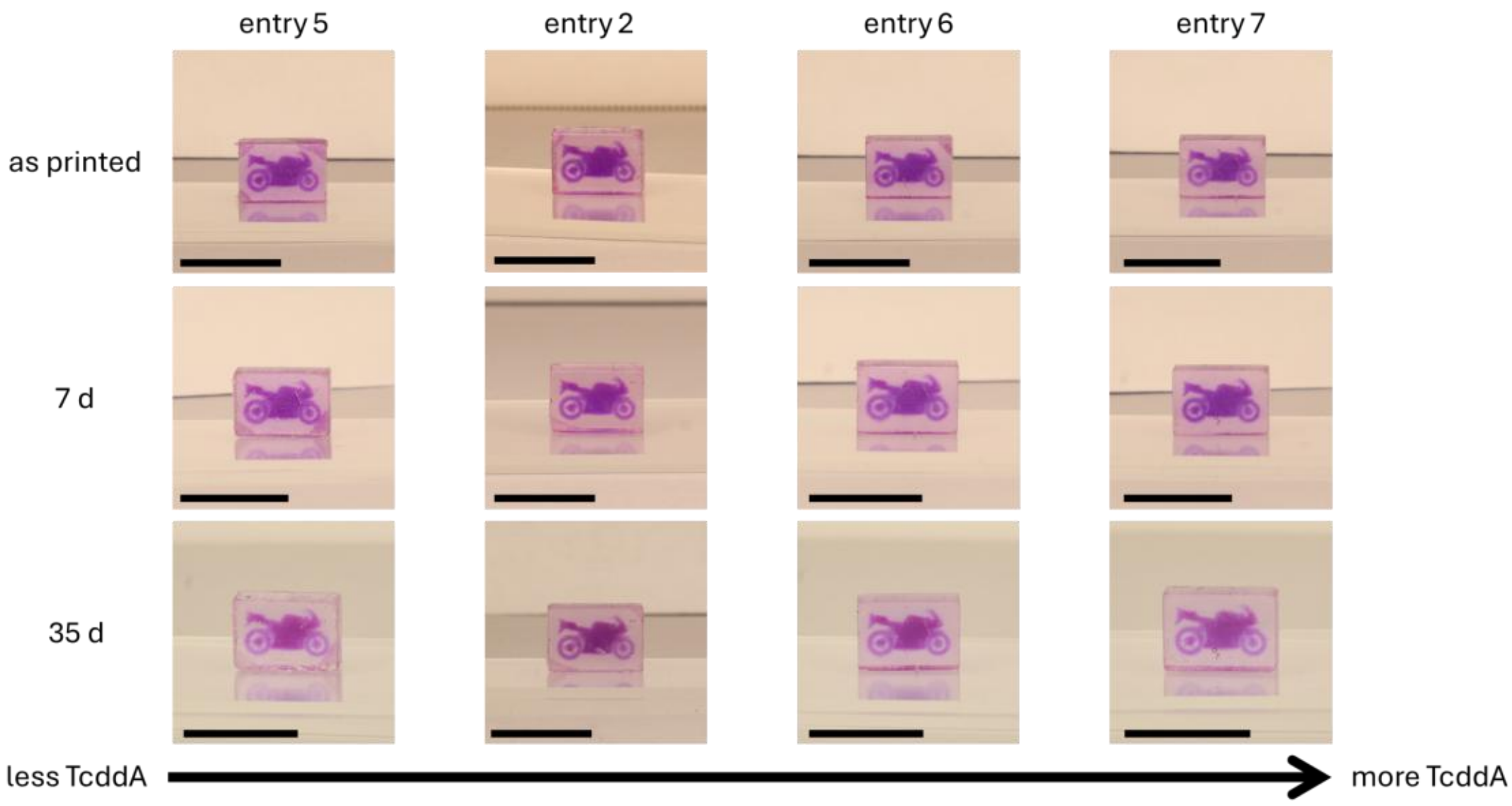

Figure S9: Images of programmed structures printed from ink formulations listed under entry 2, 5, 6 and 7 in **Table S2** are shown directly after the printing, after 7 days and after 35 days. The samples were stored in the dark at -18 °C (scale bars = 1 cm).

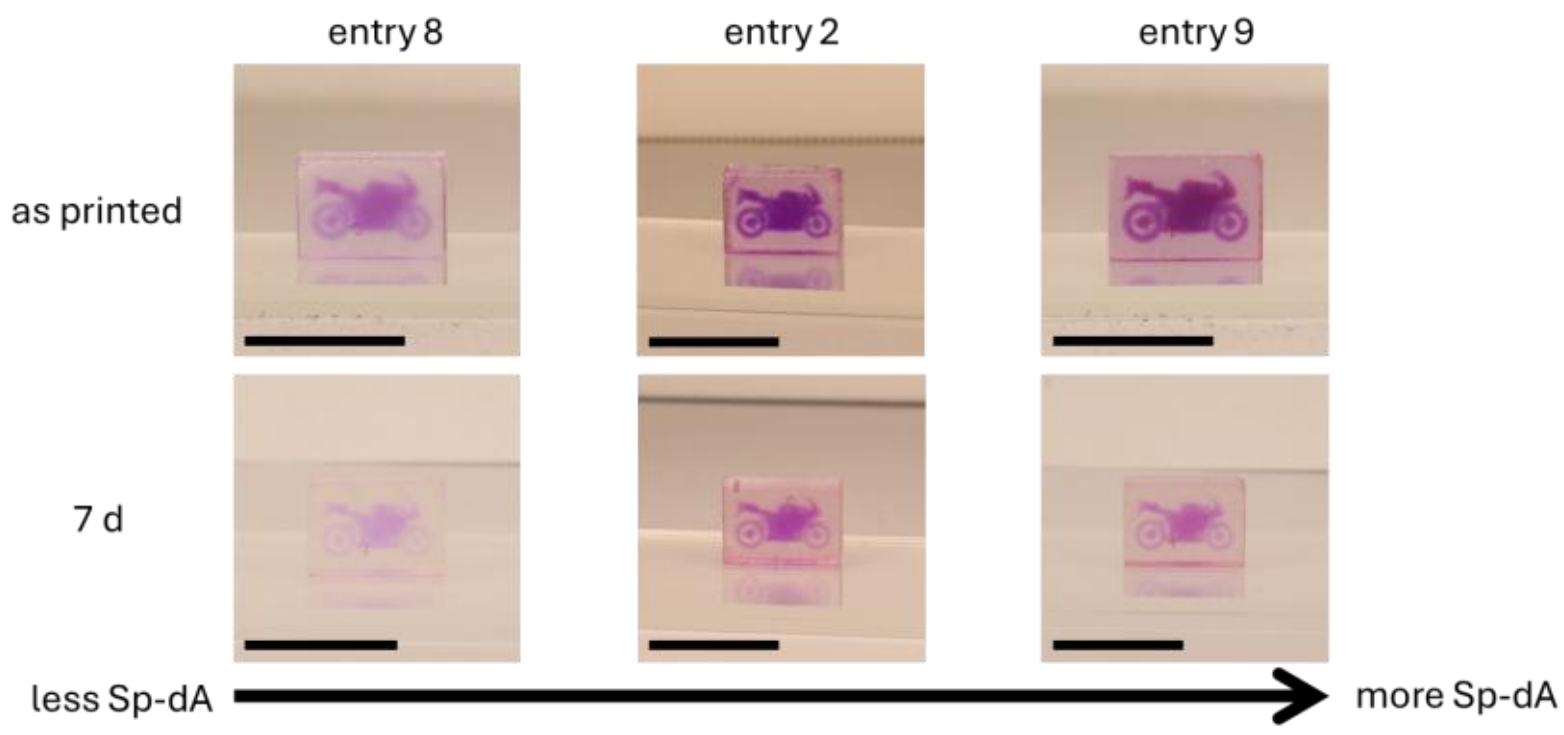

Figure S10: Images of programmed structures printed from ink formulations listed under entry 2, 8 and 9 in **Table S2** are shown directly after the printing and after 7 days. The samples were stored in the dark at 20 °C (scale bars = 1 cm).

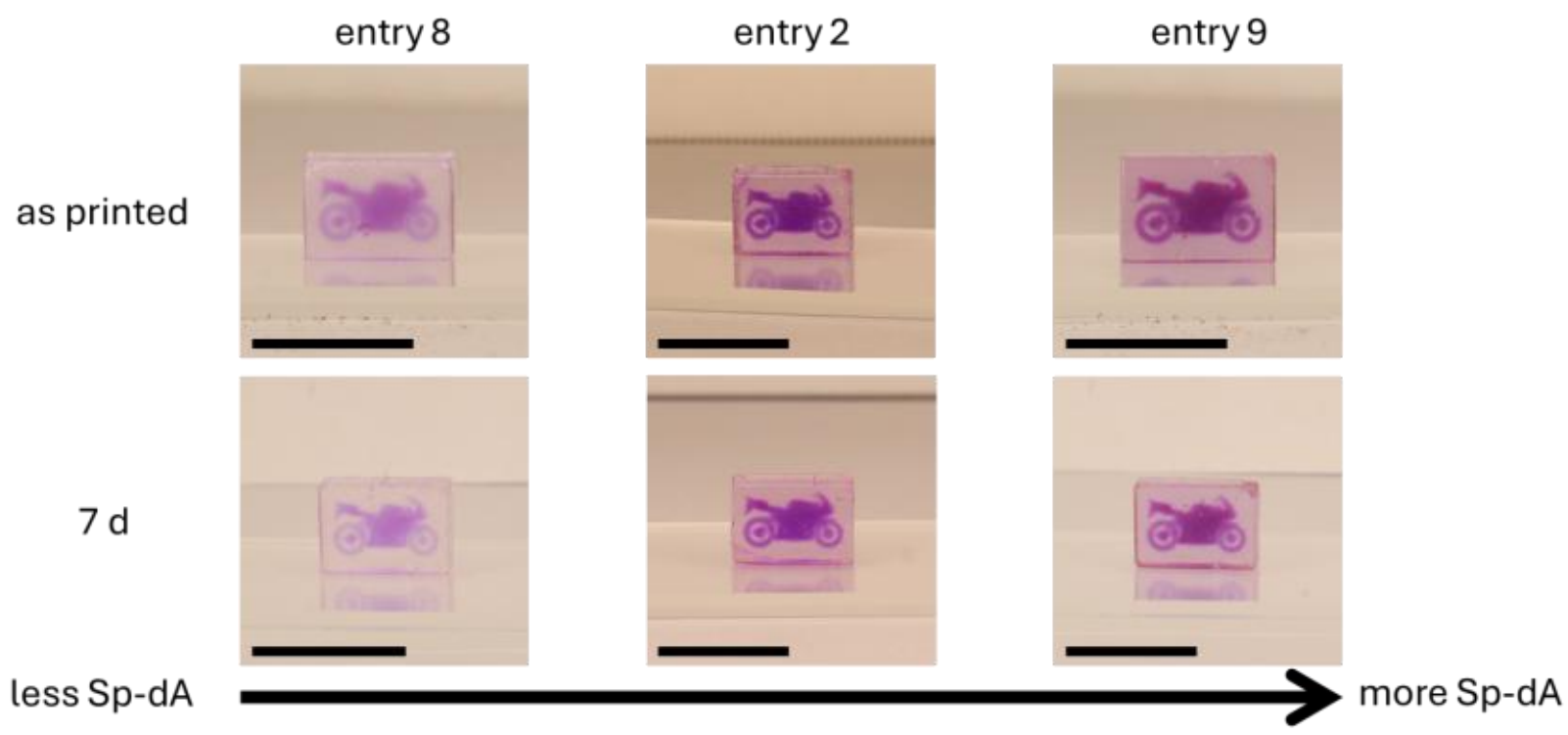

Figure S11: Images of programmed structures printed from ink formulations listed under entry 2, 8 and 9 in **Table S2** are shown directly after the printing and after 7 days. The samples were stored in the dark at -18 °C (scale bars = 1 cm).

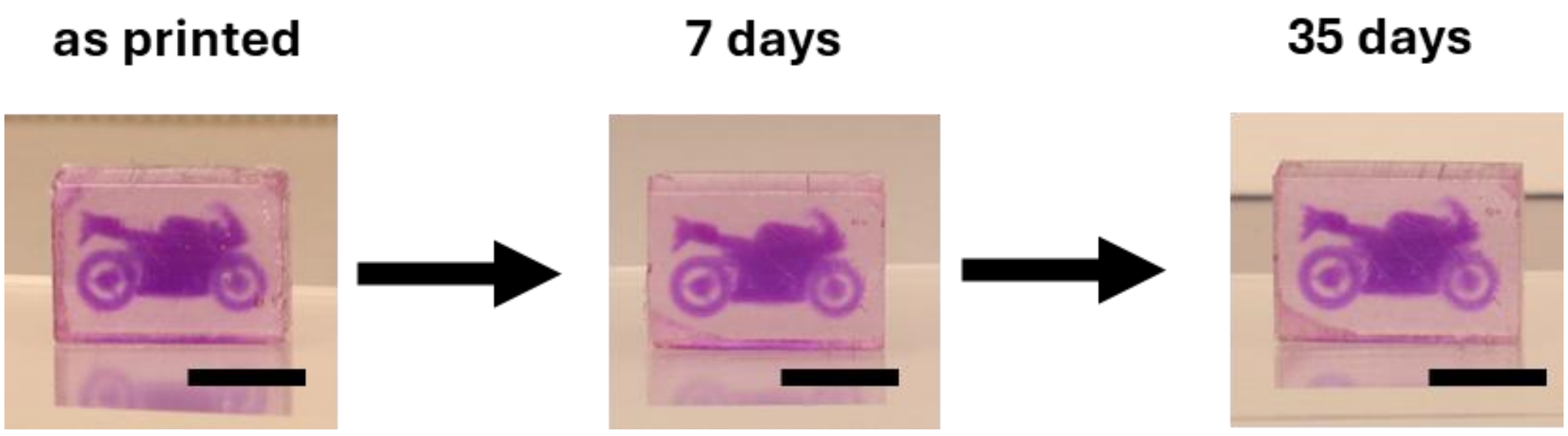

Figure S12: Images of 3D printed cuboid structures contain a programmed motorcycle from the optimized ink formulation, which were stored in the dark at -18 °C for up to 35 days (scale bars = 5mm). RGB values were determined and further highlight the stability of the encoded information: Pattern as printed (107, 25, 111) after 7 days (131, 40, 140) and after 35 days (119, 40 ,108).

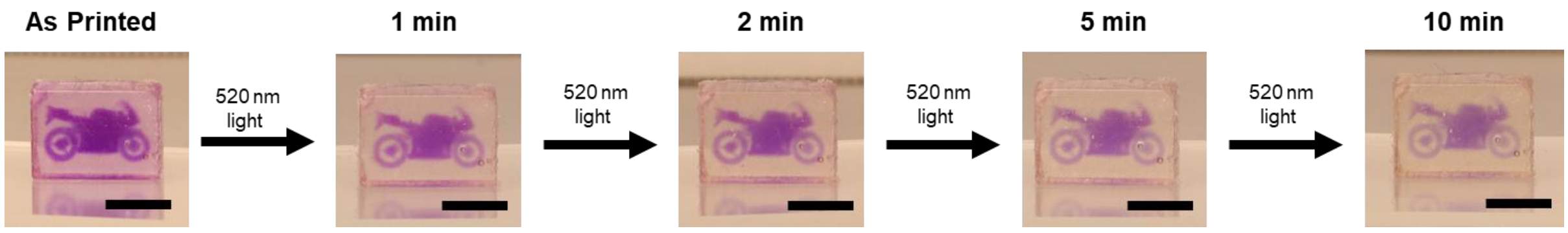

Figure S13: The stability of the programmed information in a sample printed from the optimized ink formulation, was evaluated under irradiation of a bright 520 nm wavelength LED (71 mW cm^{-2}) for 1, 2, 5, and 10 min. Even under these harsh conditions, only partial isomerization from Mc to Sp was detected, thus the information stayed well readable and can still be read out (scale bars = 5 mm).

6. Resolution Tests

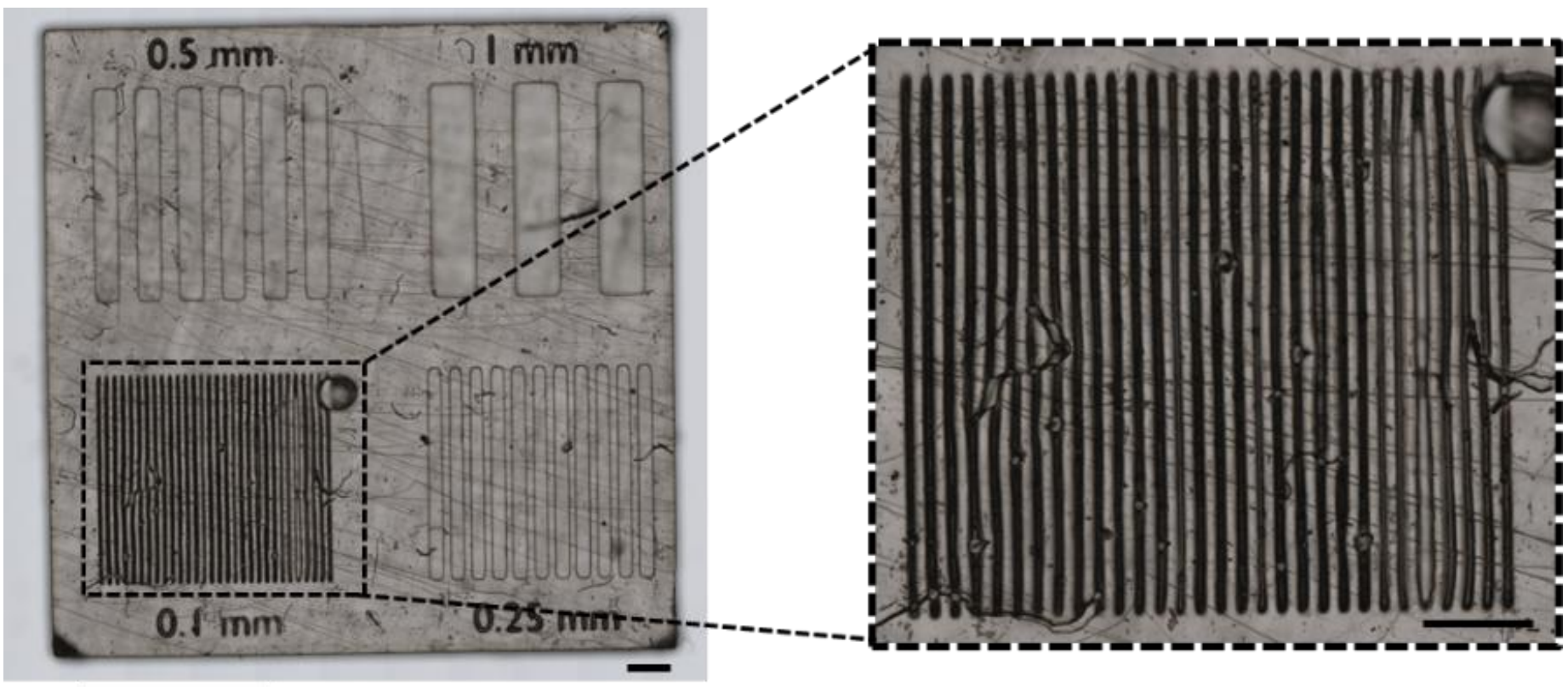

Figure S14: The image of the 3D DLP printed cuboid from the optimized ink formulation containing only Sp-dA to determine the minimal resolution (scale bars = 1 mm).

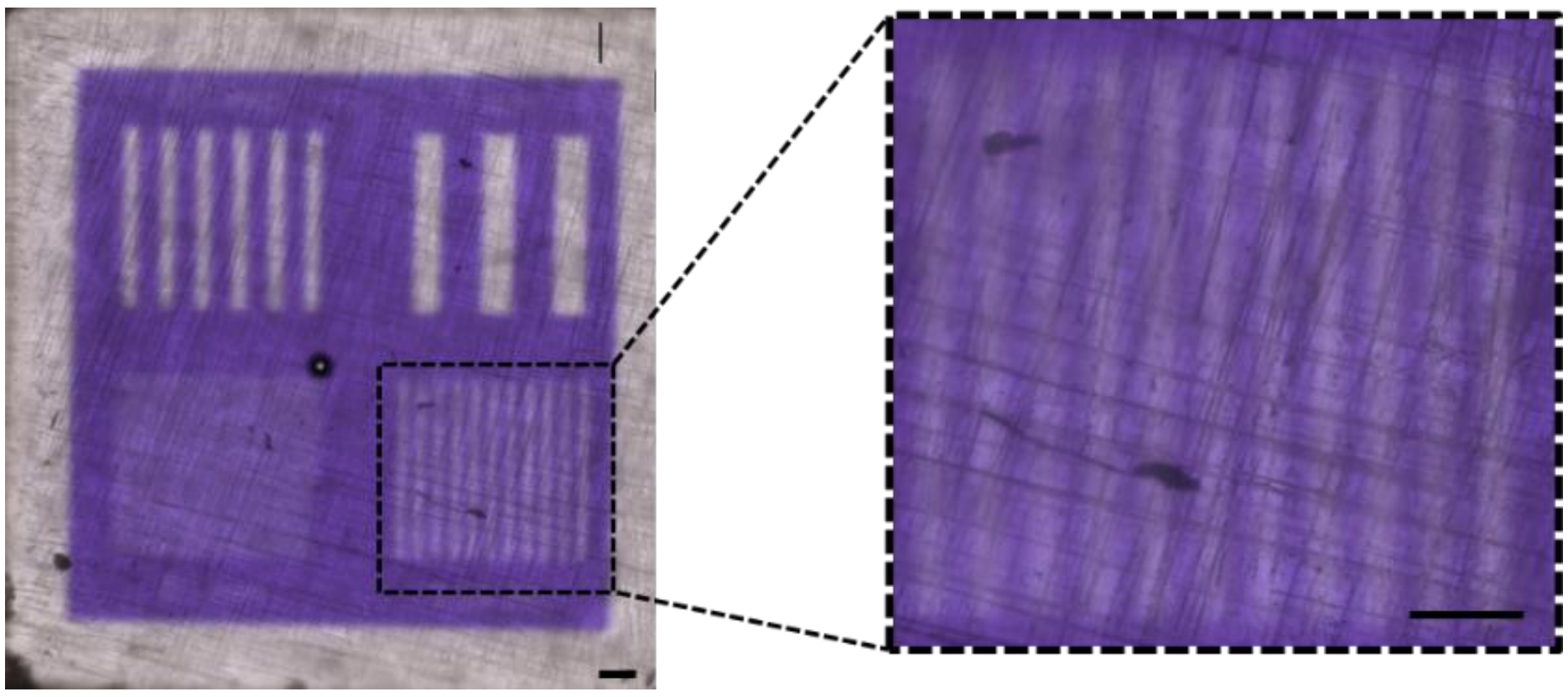

Figure S15: Image to determine the minimal resolution of the programmed information inside a 3D printed structure from the optimized ink formulation, containing only Sp-dA (scale bars = 1 mm).

7. Sectioning pH-Responsive Colored Sample

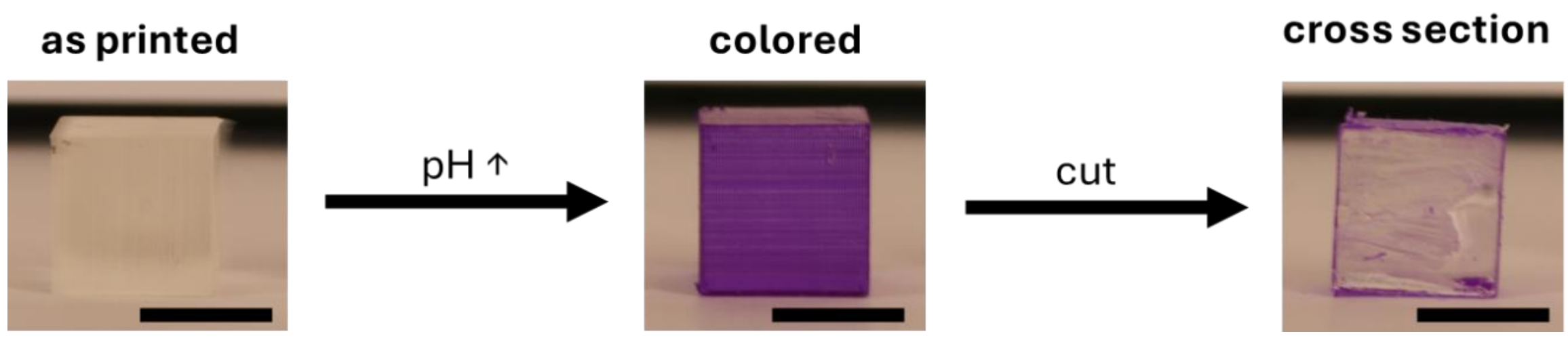

Figure S16: The cuboid printed from the only $PPAAH_2$ containing ink formulation is shown, as printed, after being immersed in a 0.5 M TBD acetone solution, and after both sides of the cuboid were cut off. It was observed that only the surface near area is subjected to deprotonation of the $PPAAH_2$ to the colored $PPAA^{-2}$ (scale bars = 5 mm).

8. UV/Vis Spectra

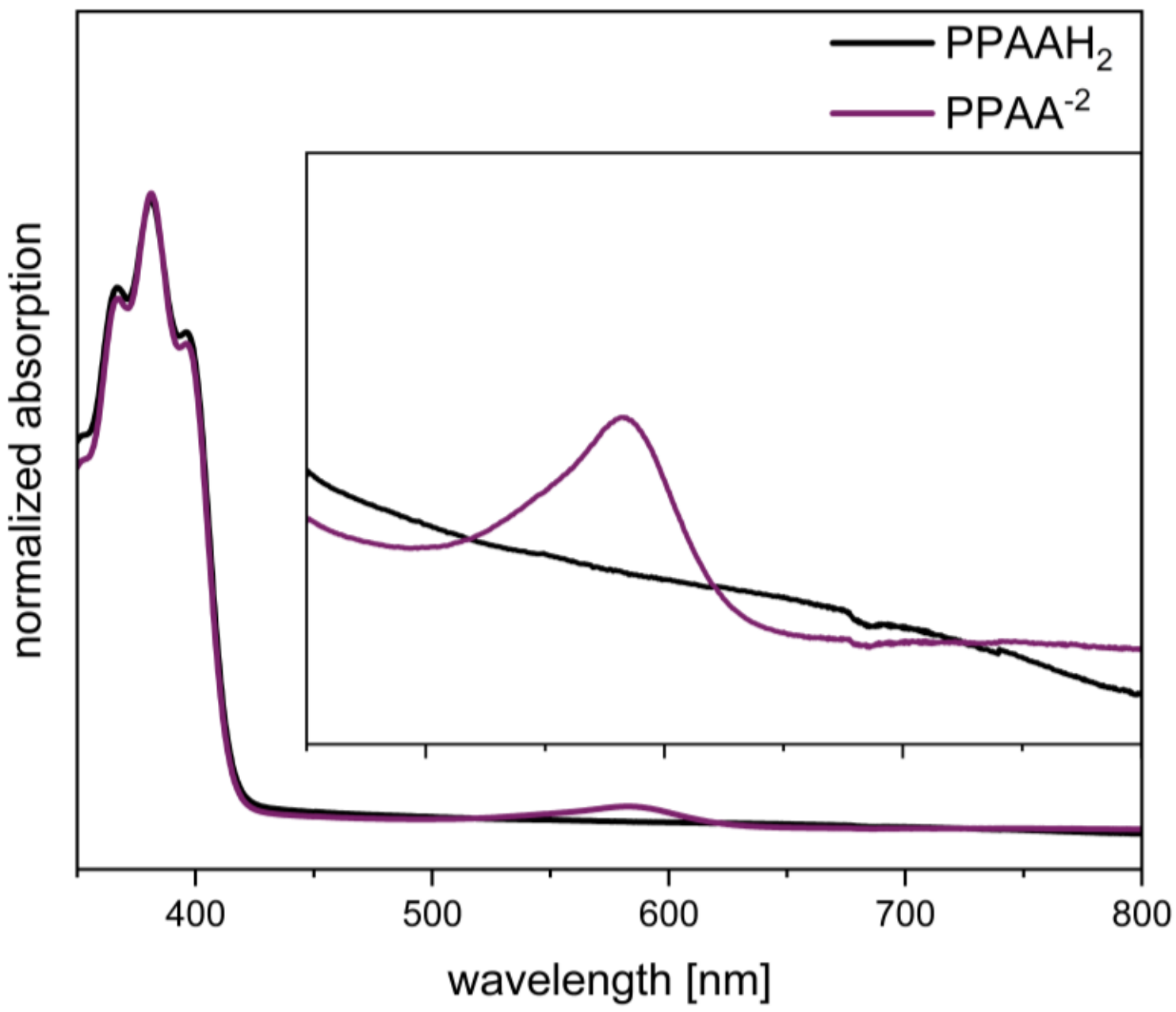

Figure S17: UV/Vis spectrum of a film cured from ink formulation containing only $PPAAH_2$ and no Sp-dA.

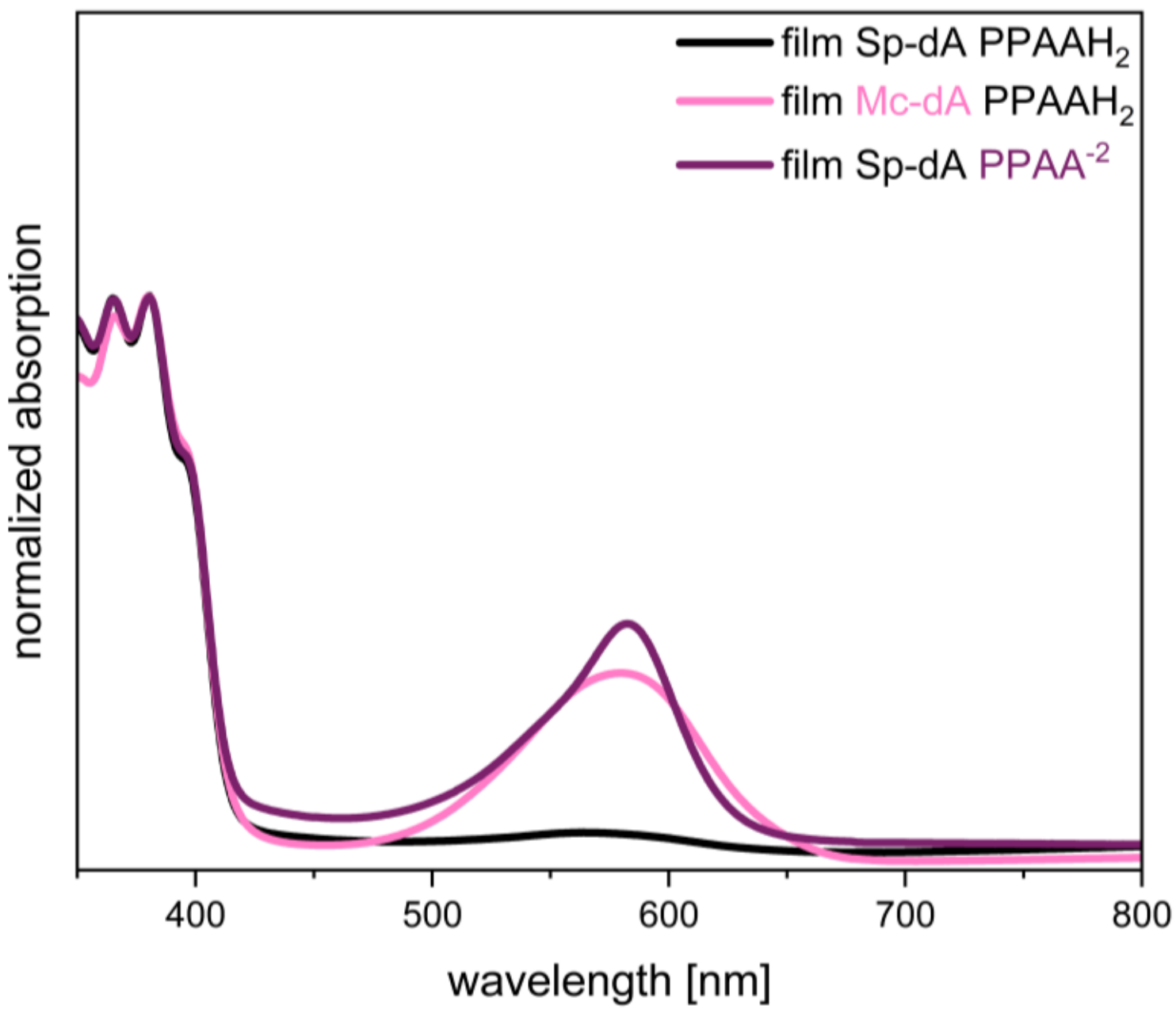

Figure S18: UV/Vis spectrum of a film cured from ink formulation containing Sp-dA and 5.0wt% $PPAAH_2$.

9. Optimization of the Final Ink Formulation

Table S3: Ink formulations with increasing $PPAAH_2$ content to systematically investigate the encryption potential of the programmed information.

entry	Sp-dA	$PPAAH_2$	IsboA	TcddA	HEA	TPO
A	0.2 wt%	1.0 wt%	54.1 wt%	40.0 wt%	1.8 wt%	2.9 wt%
B	0.2 wt%	3.0 wt%	53.3 wt%	38.8 wt%	1.7 wt%	2.9 wt%
C	0.2 wt%	5.0 wt%	52.3 wt%	38.0 wt%	1.7 wt%	2.8 wt%

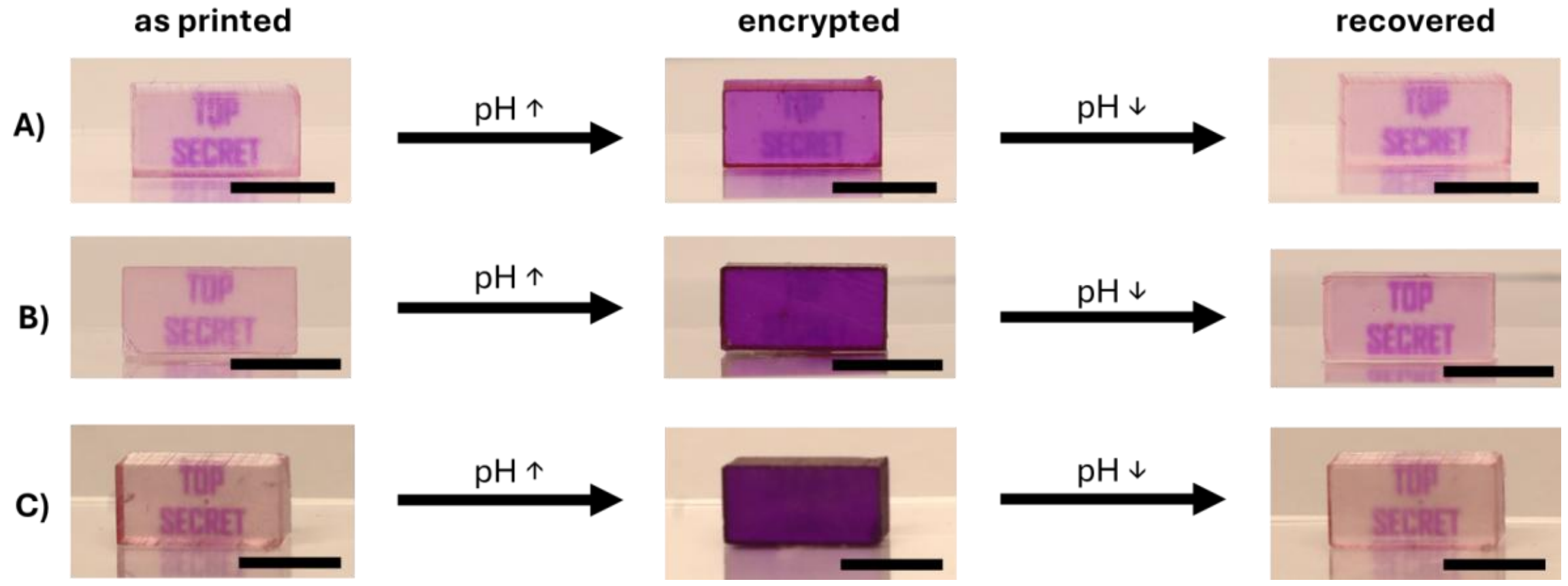

Figure S19: Samples DLP printed from ink formulations listed in **Table S3**. Images were taken directly after the printing, after immersing the structure in 0.5 M solution of TBD in acetone for 60 s, and after immersing the sample in 0.5 M solution of AcOH in acetone for 30 s (scale bars = 5 mm).

10. Repeatable pH-Response

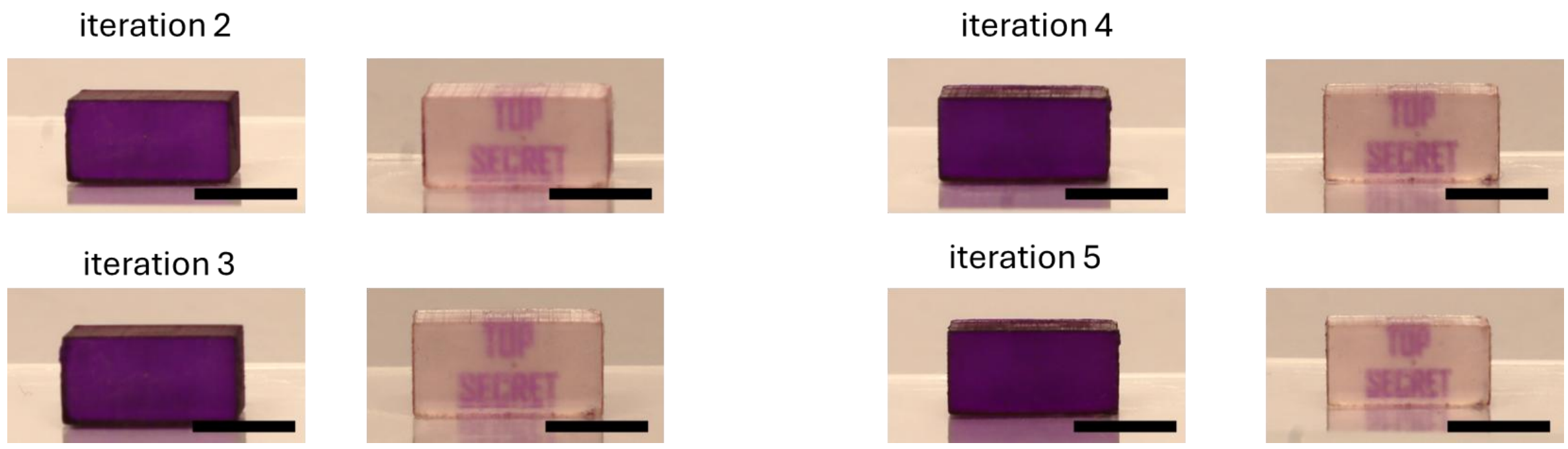

Figure S20: The images of the 2, 3, 4, and 5 iterations of encrypting the sample and recovering the programmed information (scale bars = 5 mm).

11. ^{1}H NMR Spectra

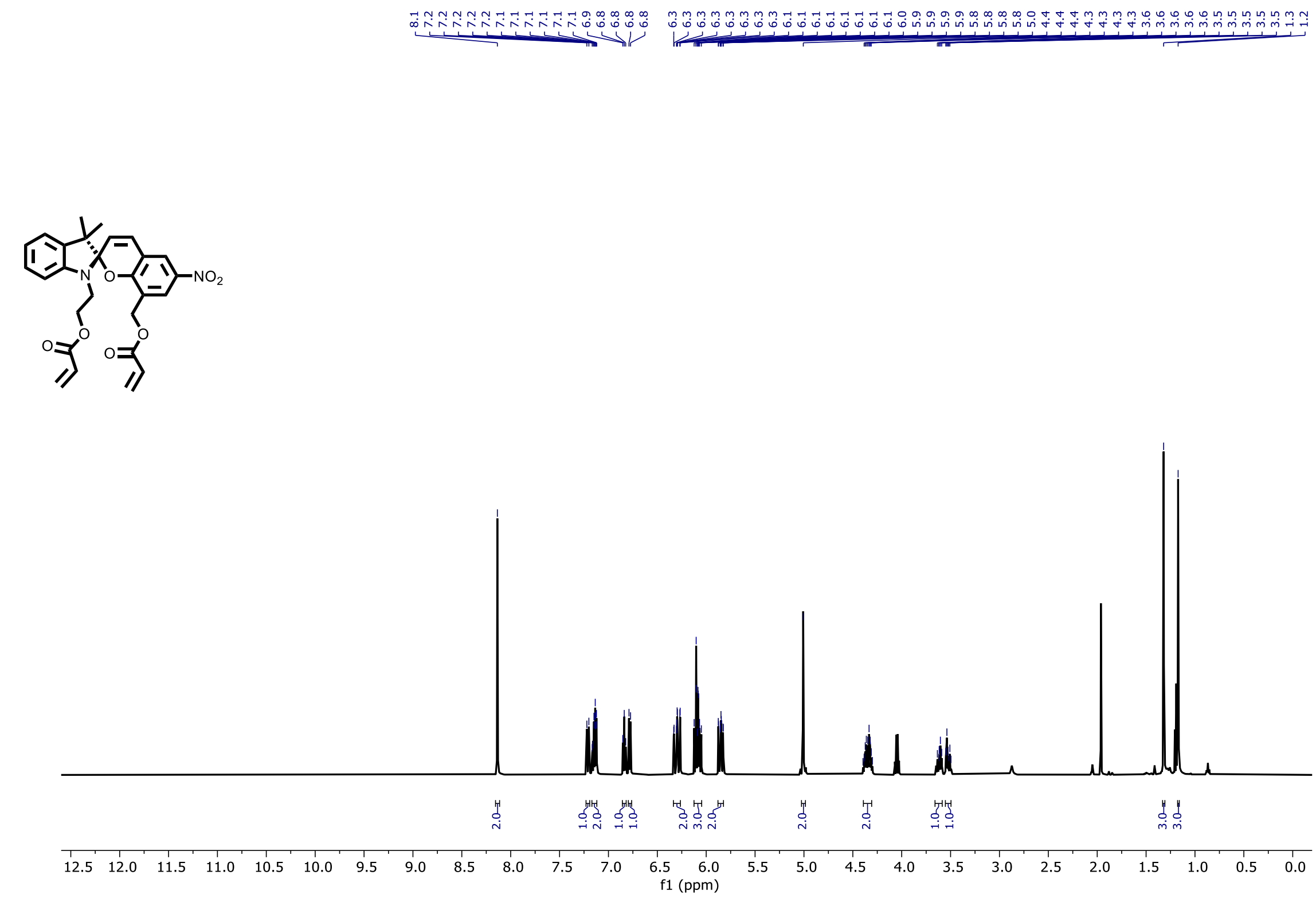

Figure S21: ^{1}H NMR spectrum of **Sp-dA** (500 MHz, acetone-d_6).

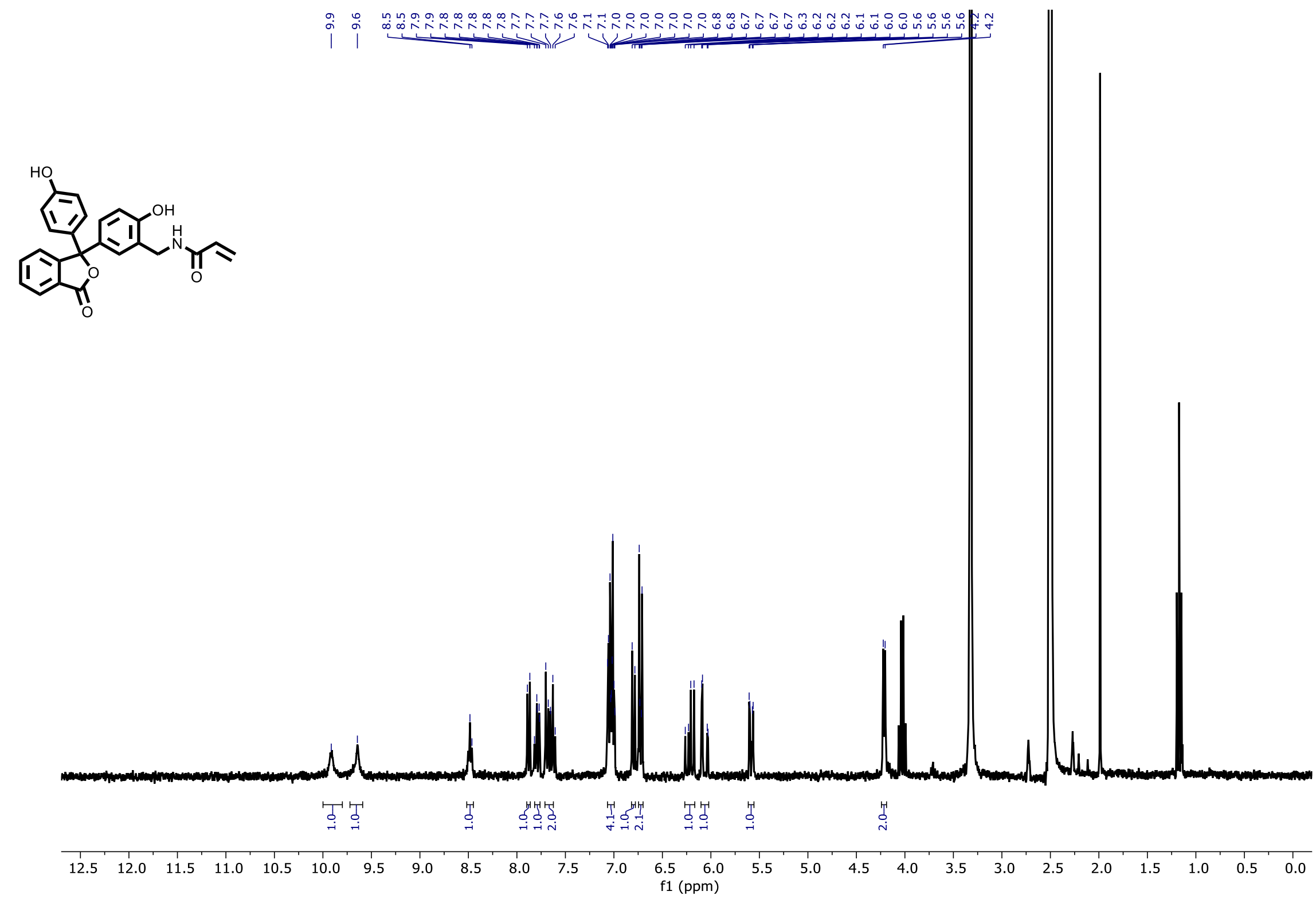

Figure S22: ^{1}H NMR spectrum of PPAAH$_2$ (301 MHz, DMSO-d_6).

12. References

[1] P. F. Jacobs, *Rapid Prototyping & Manufacturing: Fundamentals of Stereolithography*, Society of Manufacturing Engineering, Dearborn, MI, 1992.

[2] F. Kröger, C. A. Spiegel and E. Blasco, "3D DLP printed mechanochromic materials for visual signaling," *Materials Advances* **7**, (2026): 3268.

[3] C. Fleischmann, J. Cheng, M. Tabatabai and H. Ritter, "Extended Applicability of Classical Phenolphthalein: Color Changing Polymeric Materials Derived from pH-Sensitive Acrylated Phenolphthalein Derivatives," *Macromolecules* **45**, (2012): 5343.

[4] C. Fleischmann, H. Wöhlk and H. Ritter, "End group functionalization of poly(ethylene glycol) with phenolphthalein: towards star-shaped polymers based on supramolecular interactions," *Beilstein Journal of Organic Chemistry* **10**, (2014): 2263.
